# Additional Effect of Exercise to Intermittent Fasting on Body Composition and Cardiometabolic Health in Adults With Overweight/obesity: A Systematic Review and Meta-analysis

**DOI:** 10.1007/s13679-025-00645-9

**Published:** 2025-06-19

**Authors:** Zi-han Dai, Ke-wen Wan, Po-san Wong, Wendy Yajun Huang, Angus Pak-hung Yu, Stephen Heung-sang Wong

**Affiliations:** 1 Department of Sports Science and Physical Education, The Chinese University of Hong Kong, Shatin, Hong Kong China; 2 Academy of Wellness and Human Development, Hong Kong Baptist University, Kowloon Tong, Hong Kong China; 3 Dr. Stephen Hui Research Centre for Physical Recreation and Wellness, Hong Kong Baptist University, Kowloon Tong, Hong Kong China

**Keywords:** Intermittent fasting, Exercise, Overweight/Obesity, Body composition, Cardiometabolic health, Meta-analysis

## Abstract

**Objective:**

The purpose of this study is to evaluate the additional effect of exercise to intermittent fasting (IF) on body composition and cardiometabolic health in adults with overweight/obesity.

**Method:**

Relevant studies were identified through a systematic search across five databases. A random-effects meta-analysis was conducted on outcomes including body composition and cardiometabolic health markers, to compare the effect of combining IF with exercise against IF alone. The certainty of the evidence was evaluated using the GRADE approach, while the quality of the included studies was assessed using the revised Cochrane risk-of-bias tool and the TESTEX scale.

**Result:**

In total, twelve studies including 616 participants (Age: 40 ± 9 years; BMI: 33.6 ± 4.8 kg/m^2^; 87.3% female) were included in this systematic review and meta-analysis. The combined intervention was more effective, leading to greater reductions in fat mass (-0.93 kg [95% CI: -1.69, -0.18]) and waist circumference (-2.51 cm [95% CI: -3.70, -1.32]) when compared to IF alone. Cardiometabolic health parameters also showed greater improvements, with decreased insulin (-3.1uIU/ml [95%CI: -4.25; -1.95]), HOMA-IR (-0.57 [95%CI: -0.83; -0.31]), LDL (-10.67 mg/dl [95%CI: -20; -1.35]), resting heart rate (-2.68 bpm [95%CI: -4.71; -0.64]), along with enhanced cardiorespiratory fitness (VO_2 max_:1.92 ml/kg/min [95%CI: 0.32; 3.52]). For the remaining outcome variables, no statistically meaningful differences emerged between the groups.

**Conclusion:**

The potential benefits of incorporating exercise into IF for adults with overweight/obesity, particularly in terms of body composition, glycemic control, and cardiorespiratory fitness, appear promising. Nevertheless, given the limited amount of data, future investigations are essential to strengthen the evidence base and investigate the influence of specific exercise and IF types in enhancing treatment outcomes.

**Registry and registry number for systematic reviews or meta-analyses:** CRD42024550753.

**Supplementary Information:**

The online version contains supplementary material available at 10.1007/s13679-025-00645-9.

## Introduction

Overweight and obesity are major public health concerns worldwide. The global prevalence of excess body weight has increased substantially over the past few decades and is expected to continue to rise [1, 2]. According to data from the World Health Organization, in 2022, more than 2.5 billion adults were living with excess weight, and of these, 890 million were living with obesity. This trend is particularly alarming, as living with obesity is linked to cardiometabolic risk factors, increasing the risk of dyslipidemia, hypertension, and type 2 diabetes [3, 4]. Given the obesity epidemic, it is crucial to prevent and manage excess body weight from early life through adulthood using primary care such as diet modification, physical activity, and behavioral changes [5, 6].

Intermittent fasting (IF) has emerged as a promising dietary strategy for weight management and improving metabolic health in individuals with overweight and obesity [7, 8]. Apart from Ramadan intermittent fasting, which follows a religious fasting pattern, various IF protocols are employed outside religious contexts. These include diverse eating patterns such as time-restricted eating (TRE), intermittent dry fasting, specific fasting days per week, and alternate day (modified) fasting (ADF) [9]. Characterized by alternating cycles of eating and fasting, IF has been associated with numerous health benefits, including weight loss [10, 11], improved insulin sensitivity [12–14], and reduced inflammation [15–17]. While dietary strategy focuses on energy intake, exercise, which increases energy expenditure, plays an essential role, and offers numerous health benefits in adults with overweight/obesity [18]. Exercise is globally recognized for its popularity, accessibility, and established health benefits across diverse populations, including individuals with various health conditions although through different modalities [19–21]. It plays a significant role in promoting overall health and addressing metabolic disorders [22], improved glycolipid profiles [23, 24], reduced blood pressure [25], and enhanced cardiorespiratory fitness (CRF) [26]. CRF is a key indicator of cardiovascular health [27], reflects the body’s efficiency in oxygen usage during physical activity, and is associated with lower risks of chronic diseases and better metabolic functioning [28]. Given these marked benefits, exercise appears to be a valuable addition to dietary interventions, such as IF, for managing excess body weight and its associated complications. There has been growing interest in exploring the synergistic effects of combining IF with exercise. This combination has the potential to improve body composition and enhance training adaptations, though its impact on lean mass and cardiometabolic markers remains unclear [29]. Exercise plays a crucial role in supporting endocrine health by regulating metabolic hormones like insulin and cortisol, which may amplify the benefits of IF by improving insulin sensitivity and reducing stress-related hormonal responses [30]. Understanding whether exercise provides significant additional benefits when combined with IF is crucial, as it would allow clinicians and health professionals to tailor interventions more effectively for individuals with overweight or obesity.

Existing literature indicates that adding exercise to hypocaloric diets enhances cardiovascular fitness but no effects in body composition or glycemic control in adults with overweight/obesity and type 2 diabetes [31]. While previous studies have explored the combined effects of IF and exercise, one was a narrative review reporting equivocal evidence [29], another was a network meta-analysis focusing on weight loss outcomes [32], and others were meta-analyses that quantitatively analyzed the data but primarily compared IF combined with exercise to non-fasting diets with exercise [33, 34]. However, there remains limited synthesized evidence on the additional benefits of combining exercise with IF for body composition and cardiometabolic health in adults with overweight/obesity. This meta-analysis aims to evaluate randomized controlled trials (RCTs) that assessed the additional effect of incorporating exercise into IF protocols on body composition and cardiometabolic health in adults with overweight/obesity. By synthesizing data from multiple studies, we seek to determine whether this combined approach offers greater health benefits compared to IF alone, thereby providing evidence-based insights and practical guidelines for tailoring interventions to optimize clinical outcomes in this population.

## Methods

### Design

This systematic review and meta-analysis were registered at PROSPERO (registration number CRD42024550753) and conducted in accordance with the Preferred Reporting Items for Systematic Reviews and Meta-analyses (PRISMA) statement guidelines and the Cochrane Handbook of Systematic Reviews of Interventions [35].

### Search Strategy

A systematic literature search was conducted to identify studies evaluating how IF combined with exercise impacts cardiometabolic outcomes and body composition. Searches were performed within electronic databases such as PubMed, Embase, SPORTDiscus, Web of Science, and Cochrane Central Register of Controlled Trials (CENTRAL), covering all available records up to July 2024. The search strategy included various combinations of the keywords and MeSH terms: (“Intermittent fasting” OR “alternate-day fasting” OR “intermittent energy restriction” OR “intermittent calorie restriction” OR “intermittent restrictive diet” OR “periodic fasting” OR “sporadic fasting” OR “time-restricted feeding” OR “time-restricted eating” OR “5:2 diet” OR “5:2 fasting” OR “Ramadan” OR “Ramadhan” OR “time-restricted fasting” OR “periodic diet” OR “reduced meal frequency” OR “alternate day modified fasting” OR “modified alternate-day fasting” OR “whole day fasting”) AND (Exercise* OR aerobic* OR running OR jogging OR walk* OR hiking OR swim* OR aquatic* OR cycling OR bicycle* OR strength* OR physical activity* OR fitness OR train* OR resistance) AND (Obesity OR Obese or Overweight). Table S1 provides a detailed description of the search methods. Further relevant articles were discovered by screening the reference lists of the studies obtained via the systematic search.

### Criteria for Eligibility and Selection of Studies

After identifying studies through databases, registries, and additional sources, all records were imported into Endnote 20 software. All titles and abstracts underwent independent screening by two authors (ZD and KW), followed by full-text assessment for final study inclusion. Any differences identified by the two reviewers were resolved by consulting a third reviewer (PW) and engaging in discussion until agreement was achieved. Studies meeting the following criteria were included: 1) adults with overweight and/or obesity, BMI ≥ 23 kg/m^2^ (Asians study [36] BMI ≥ 23 kg/m^2^); 2) Randomized controlled trials with a matched comparison between IF combined with exercise (IF + EX) and IF alone; 3) Evaluation of body composition and/or cardiometabolic outcomes, with body mass, fat mass, fat-free mass, body mass index (BMI), visceral fat mass, waist circumference (WC), fasting glucose, insulin, homeostatic model assessment for insulin resistance (HOMA-IR), low-density lipoprotein cholesterol (LDL), high-density lipoprotein cholesterol (HDL), total cholesterol (TC), triglycerides (TG), systolic blood pressure (SBP), diastolic blood pressure (DBP), resting heart rate, and maximal oxygen uptake (VO_2 max_), leptin and adiponectin. The searches were restricted to studies involving humans, with the availability of full-text articles published exclusively in English; however, no limitations were imposed regarding the date of publication.

### Data Extraction

Data extraction from the selected studies was conducted by a single author (ZD) using an electronic spreadsheet (Excel, 2023) based on the specified study characteristics, including study details (first author, publication year, country, participant characteristics, study design), intervention specifics encompassing types of IF and exercise, comparator details, intervention duration, and outcomes. The dataset included the number of participants, mean differences, and standard deviations (SD) of the outcomes from baseline to endpoint for both the intervention and control groups. These final data were prepared for analysis in the R statistical software (version 4.4.1). When obtaining complete data was not feasible, we employed a correlation coefficient (Corr) to calculate the SD of the pre-post change (SD_change_). The Corr was derived based on the calculated coefficients [37]. The following formula was applied to compute the standard deviations of the SD_change_. ${\text{SD}}_{\text{change}}=\sqrt{{\text{SD}}_{\text{baseline}}^{2}+{\text{SD}}_{\text{endpoint}}^{2}-(2\times \text{Corr}\times {\text{SD}}_{\text{baseline}}\times {\text{SD}}_{\text{endpoint}})}$. All outcome measures were converted into consistent units, such as fasting glucose (mg/dl), TC (mg/dl), TG (mg/dl). In cases where change values were not reported, we reached out to the corresponding authors of the articles to obtain the necessary data. When sufficient data was unavailable, values were retrieved from graphs utilizing the WebPlotDigitizer software. All extracted information was independently verified for correctness by another author (KW).

### Study Risk of Bias Assessment

Two reviewers (ZD and PW) independently assessed the risk of bias using the revised Cochrane Risk of Bias tool (RoB 2) [38]. Discrepancies in their assessments were resolved through discussions with an additional author (KW) until a consensus was achieved. Five domains were assessed to evaluate the quality of the included RCTs, including bias from the randomization process, deviations from intended interventions, missing outcome data, outcome measurement, and the selection of reported results. Each study was subsequently assigned an overall risk of bias classification as either “low risk”, “some concerns,” or “high risk,” based on these assessments.

### Certainty of Evidence

The overall certainty of evidence was evaluated using the Grading of Recommendation Assessment, Development, and Evaluation (GRADE) approach. This method evaluated the certainty of evidence across five key domains: risk of bias, inconsistency, indirectness, imprecision, and publication bias [39]. The GRADE approach was applied to evaluate all outcomes in this meta-analysis, and any author disagreements were addressed through discussion until consensus was achieved.

### Assessment of Study Quality and Reporting in Exercise

The quality of the included studies was also evaluated using the Tool for the assessment of study quality and reporting in exercise (TESTEX) scale, a tool specifically designed for assessing the quality and reporting of exercise-based studies. This evaluation covered 12 domains, encompassing both study quality and reporting components. Based on their total TESTEX scores, the studies were categorized as high quality, good quality, or low quality [40].

### Data Synthesis and Analysis

One author (ZD) performed data synthesis and analysis, and meta-analyses were completed utilizing R statistical software (version 4.4.1) with the metafor package (version 7.0–0) when data were available from a minimum of two reports. These analyses involved calculating mean differences (MD) and standardized mean differences (SMD) along with 95% confidence intervals (CI), to assess and compare the effects of IF + EX with IF alone on the outcomes. Random-effect models were utilized for calculations, considering potential heterogeneity stemming from clinical or methodological variations that might have impacted the results. Heterogeneity was assessed using the I-squared (I^2^) statistic, with heterogeneity considered high when I^2^ exceeded 50%. Prediction intervals (PI) were reported to illustrate the degree of heterogeneity in forest plots of random-effects meta-analyses [41]. For analyses with I^2^ > 50%, sensitivity analyses were performed by systematically excluding one study at a time to determine whether any specific study contributed significantly to the heterogeneity. To delve deeper into the effects of the intervention on body composition and cardiometabolic outcomes, subgroup analyses were conducted based on the participant’s age, sex, intervention duration, IF type, and EX type. Furthermore, potential publication bias was assessed using funnel plots and Egger’s regression analysis, provided the meta-analysis included at least ten studies. A P-value of < 0.05 was considered indicative of publication bias.

## Results

### Study Selection

**Fig. 1:**
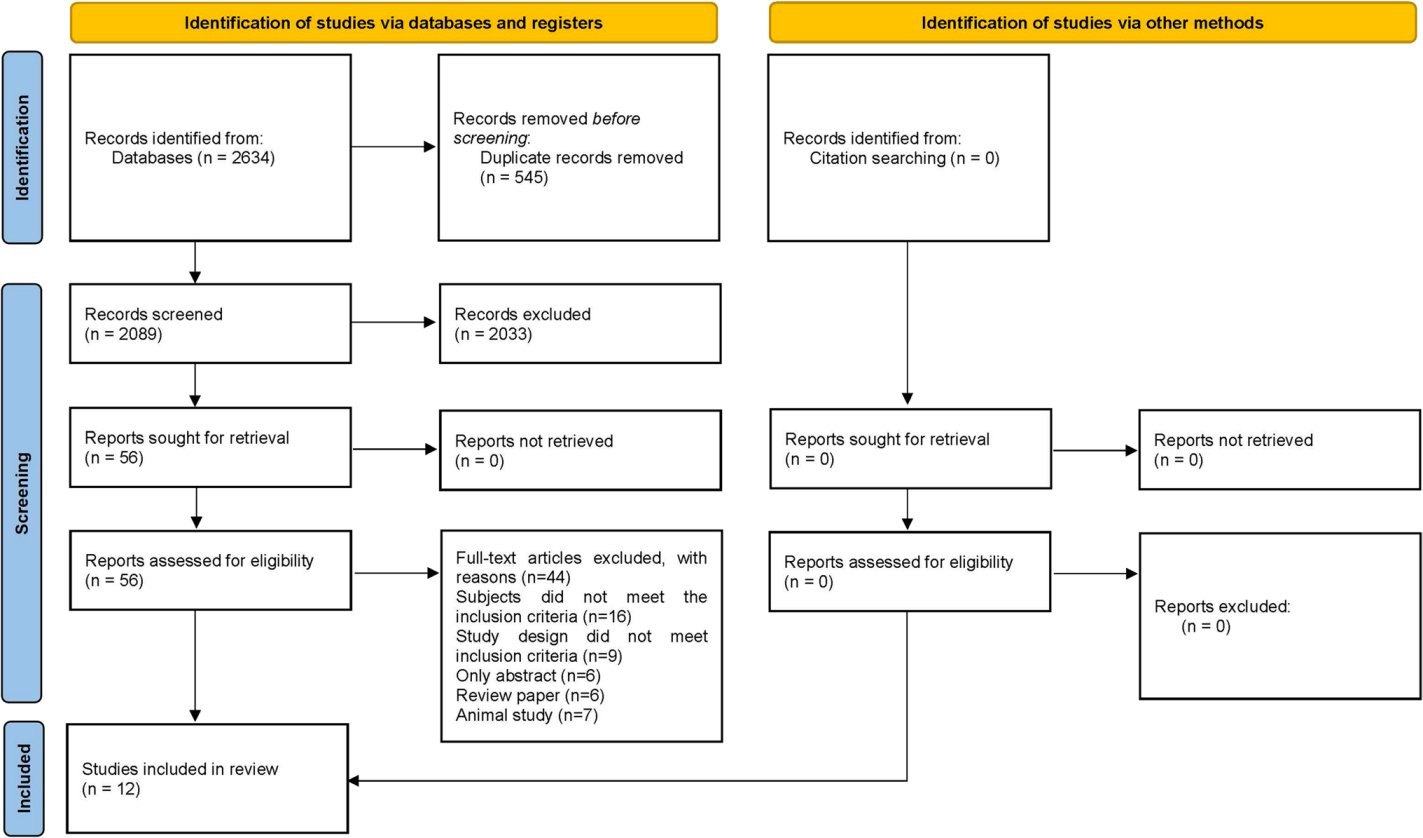
Flowchart of study selection

A total of 2,634 articles were initially retrieved through the database searches. Detailed records from each of the five databases are provided in Table S1. After deduplicating and screening titles and abstracts, 56 full-text papers were evaluated for eligibility. Ultimately, 12 articles derived from 10 studies met the criteria for inclusion in the systematic review and meta-analysis [42–53]. The detailed selection procedure is illustrated clearly in the PRISMA flowchart shown in Fig. 1.

### Study Characteristics

Table 1 provides an overview of the main characteristics of the twelve RCTs selected for inclusion in the meta-analysis [42–53]. Together, these studies involved a total of 616 participants, with an overall mean age of 40 ± 9 years, a mean BMI of 33.6 ± 4.8 kg/m^2^, and a sample that was 87.3% female. Among the 12 studies, three studies were conducted in the USA [45, 46, 49], two in Tunisia [42, 52], two in Brazil [43, 44], two in Korea [47, 53], one in Thailand [51], one in Norway [50], and one in Australia [48] with a total of 616 participants (sample size ranging from 20 to 131). Notably, Bhutani et al. [45, 46] and Batitucci et al. [43, 44] contributed two articles each, reporting on different outcomes. Intervention durations varied from 4 to 16 weeks; specifically, one study lasted 4 weeks [52], one study spanned 7 weeks [50], three studies lasted 8 weeks [43, 44, 53], six studies lasted 12 weeks [42, 45–47, 49, 51], and one study lasted 16 weeks [48]. In addition, one study exclusively enrolled male participants [52], five studies recruited only female participants [42–44, 50, 51], while the remaining six studies included participants of both sexes [45–49, 53]. Exercise interventions included continuous training [45, 46, 49], concurrent training [47, 51–53], and high-intensity interval [43, 44, 48, 50]/functional training [42]. As for IF type, two studies used TRE [42, 50], five studies used ADF [45–47, 49, 53], four studies performed 5:2 diet [43, 44, 48, 51] and one study used Ramadan IF [52].

**Table 1:** The main characteristics of the included studies

Author (year)	Country	Age, years Mean ± SD	Sex (F/M)	BMI, kg/m^2^ Mean ± SD	Participants number	Duration	IG IF + EX	CG IF	Outcome examined
Ameur et al., 2024	Tunisia	32.1 ± 10.0	Female 64/0	35.0 ± 3.8	64	12 weeks	TRE + HIFT	TRE	Body mass, BMI, DBP, FFM, FG, FM, HDL, HOMA-IR, insulin, LDL, SBP, TC, TG, WC,
Batitucci et al., 2022	Brazil	32.2 ± 4.4	Female 36/0	34.0 ± 3.2	36	8 weeks	5:2 + HIIT	5:2	Body mass, BMI, WC
Batitucci et al., 2024	Brazil	32.2 ± 4.4	Female 36/0	34.0 ± 3.2	36	8 weeks	5:2 + HIIT	5:2	FFM, FM
Bhutani et al., 2013a	US	44.0 ± 13.0	Both 80/3	35.0 ± 4.6	83	12 weeks	ADF + CT	ADF	Body mass, BMI, DBP, FFM, FG, FM, HDL, HOMA-IR, RHR, insulin, LDL, SBP, TC, TG, WC
Bhutani et al., 2013b	US	44.0 ± 13.0	Both 80/3	35.0 ± 4.6	83	12 weeks	ADF + CT	ADF	Adiponectin, leptin
Cho et al., 2019	Korea	36.7 ± 7.3	Both 16/15	27.3 ± 3.4	31	12 weeks	ADF + CON	ADF	Body mass, BMI, FG, FM, HDL, HOMA-IR, insulin, LDL, TC, TG, VFM, VO_2 max_
Cooke et al., 2022	Australia	35.4 ± 8.4	Both 28/6	31.3 ± 3.5	34	16 weeks	5:2 + HIIT	5:2	Body mass, DBP, FFM, FG, FM, HDL, HOMA-IR, LDL, SBP, TC, TG, VFM, VO_2 max_, WC
Ezpeleta et al., 2023	US	44.0 ± 13.0	Both 65/15	36.0 ± 6.0	80	12 weeks	ADF + CT	ADF	Body mass, BMI, DBP, FFM, FG, FM, HDL, HOMA-IR, RHR, insulin, LDL, SBP, TC, TG, VFM, WC
Haganes et al., 2022	Norway	36.2 ± 6.2	Female 131/0	36.2 ± 6.2	131	7 weeks	TRE + HIIT	TRE	Adiponectin, Body mass, DBP, FFM, FG, FM, HDL, HOMA-IR, leptin, RHR, insulin, LDL, SBP, TC, TG, VFM, VO_2 max_
Keawtep et al., 2024	Thailand	52.9 ± 3.4	Female 92/0	29.1 ± 3.4	92	12 weeks	5:2 + CON	5:2	Adiponectin, Body mass, BMI, FFM, FG, FM, HOMA-IR, insulin, TC, TG, VO_2 max_
Maaloul et al., 2023	Tunisia	31.8 ± 7.1	Male 0/20	33.1 ± 4.2	20	4 weeks	RIF + CON	RIF	Body mass, FFM, FG, FM, HDL, LDL, TC, TG, WC
Oh et al., 2018	Korea	36.4 ± 8.1	Both 26/19	27.4 ± 3.1	45	8 weeks	ADF + CON	ADF	Body mass, BMI, DBP, FFM, FG, FM, HDL, HOMA-IR, insulin, SBP, TC, TG, WC

5:2, 5:2 diet; ADF, alternate day fasting; BMI, body mass index; CG, control group; CON, concurrent training; DBP, diastolic blood pressure; EE, endurance exercise; FFM, fat-free mass; FG, fasting glucose; FM, fat mass; HDL, high density lipoprotein cholesterol; HIFT, high intensity function training; HIIT, high intensity interval training; HOMA-IR, homeostatic model assessment for insulin resistance; IG, intervention group; LDL, low density lipoprotein cholesterol; RIF, Ramadan intermittent fasting; RHR, resting heart rate; SBP, systolic blood pressure; TC, total cholesterol; TG, triglycerides; TRE, time restricted eating; VFM, visceral fat mass; VO_2 max_, maximal oxygen uptake; WC, waist circumference

### The Effects of IF + EX Versus IF Alone on Anthropometric and Body Composition Outcomes

**Fig. 2:**
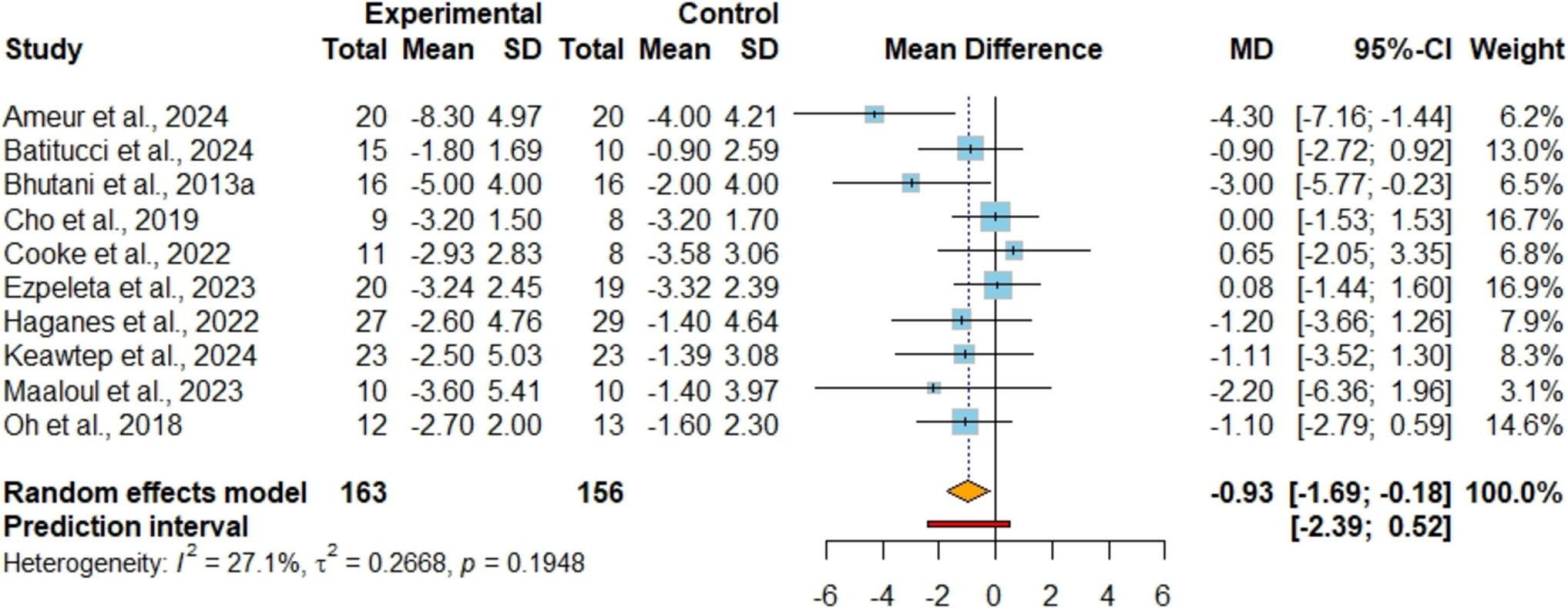
Meta-analysis of IF + EX vs. IF alone on fat mass. Values are reported as mean difference (MD)

**Fig. 3:**
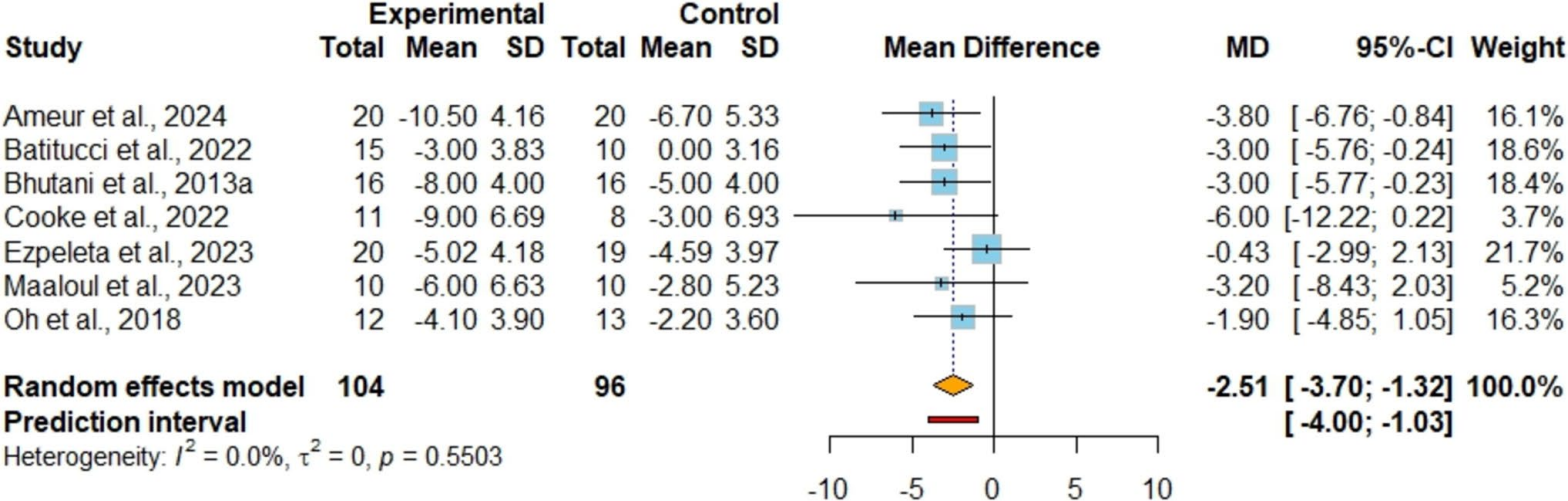
Meta-analysis of IF + EX vs. IF alone on waist circumference. (MD)

In Figure S1, ten studies [42, 43, 45, 47–53] including 319 participants, examined body mass as an outcome. The analysis showed no significant difference between the combined intervention and IF alone (MD −0.15 kg; 95%CI [−0.84, 0.54]; P = 0.67; I^2^ = 0%, 95%PI [−1.20, 0.90]), and the certainty of this evidence was rated as very low. Seven studies [42, 43, 45, 47, 49, 51, 53] involving 224 participants, investigated the impact on BMI as an outcome (Figure S2). The analysis showed no significant difference between the combined intervention and IF alone (MD −0.26 kg/m^2^; 95%CI [−0.67, 0.15]; P = 0.21; I^2^ = 0%, 95%PI [−0.78, 0.25]), and the certainty of this evidence was rated as low. In terms of fat mass, the combined strategy showed a lower level (MD −0.93 kg; 95%CI [−1.69, −0.18]; P = 0.01; I^2^ = 27%, 95%PI [−2.39, 0.52]) compared to IF alone, supported by moderate certainty of evidence from a comprehensive analysis involving 10 studies [42, 43, 45, 47–53] encompassing 319 participants (Fig. 2). For fat-free mass, the analysis of nine studies [42, 43, 45, 48–53] involving a total of 302 participants revealed no significant difference between the combined strategy and the IF alone (MD 0.75 kg; 95%CI [−0.41, 1.90]; P = 0.20; I^2^ = 86%, 95%PI [−3.04, 4.53]) (Figure S3). The certainty of this evidence was assessed as very low. Sensitivity analyses indicated that eliminating heterogeneity, notably by excluding the study by Ameur et al. [42], did not alter the statistical significance. In the analysis of WC, seven studies [42, 43, 45, 48, 49, 52, 53] involving 200 participants, with low certainty of evidence (Fig. 3), indicated that the combined strategy led to a lower reduction in WC (MD −2.51 cm; 95%CI [−3.70, −1.32]; P < 0.001; I^2^ = 0%, 95%PI [−4.00, −1.03]). Regarding the assessment of visceral fat from four studies [47–50] encompassing 131 participants (Figure S4), it was found that there was no significant difference between the combined strategy and the control group (SMD −0.20; 95%CI [−0.55, 0.15]; P = 0.26; I^2^ = 6%, 95%PI [−0.76, 0.36]), supported by evidence of low certainty.

### The Effects of IF + EX Versus IF Alone on Glycemic Outcomes

**Fig. 4:**
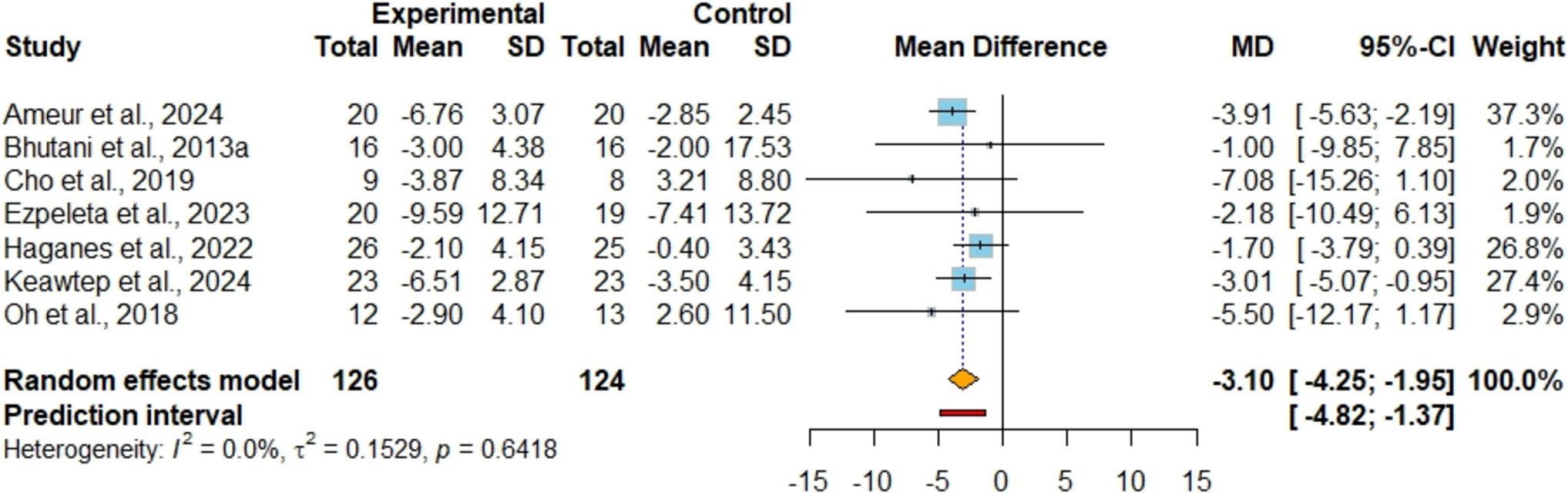
Meta-analysis of IF + EX vs. IF alone on insulin. (MD)

**Fig. 5:**
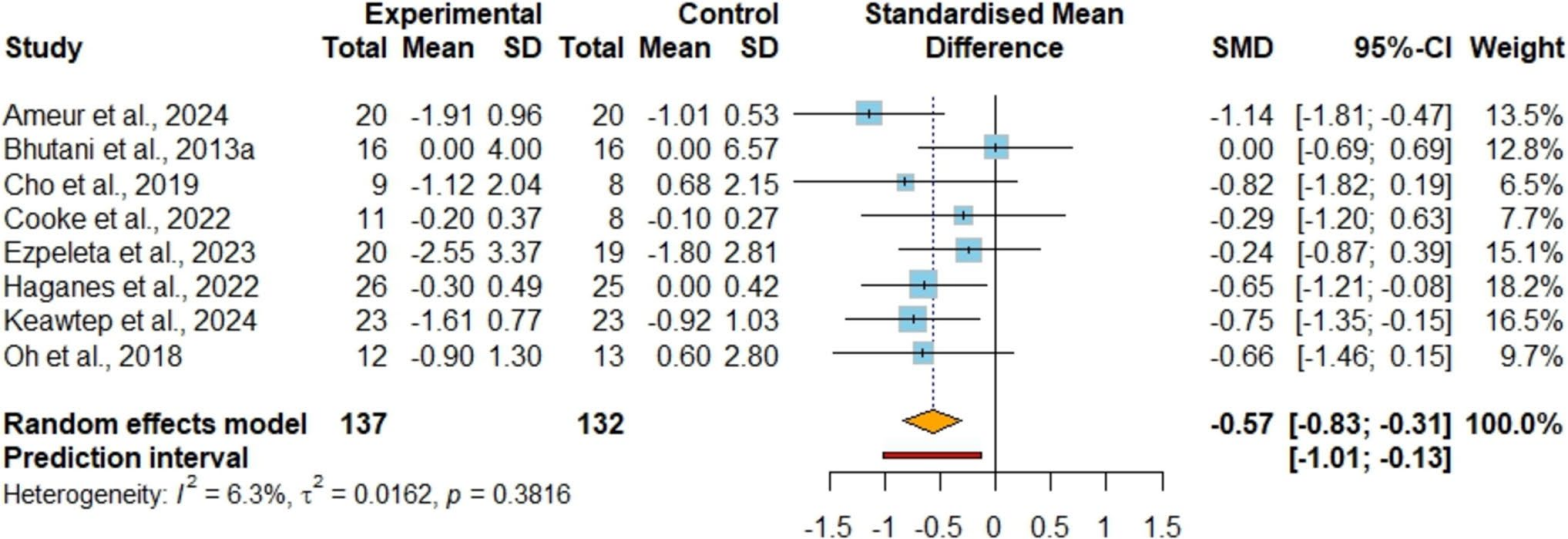
Meta-analysis of IF + EX vs. IF alone on HOMA-IR. Values are reported as standardized mean differences (SMD)

In the analysis of fasting glucose levels across nine studies [42, 45, 47–53] involving 293 participants (Figure S5), no significant difference was observed between the combined strategy and the control group (MD −1.92 mg/dl; 95%CI [−4.47, 0.62]; P = 0.14; I^2^ = 51%, 95%PI [−8.73, 4.89]), supported by evidence of very low certainty. Sensitivity analysis indicated that removing the study conducted by Maaloul et al. [52] decreased heterogeneity to 10%, although it had no impact on the statistical significance of the results. Insulin levels were assessed as an outcome in seven studies [42, 45, 47, 49–51, 53], encompassing a total of 250 participants (Fig. 4). It was observed that the combined strategy showed lower insulin (MD −3.10 uIU/ml; 95%CI [−4.25, −1.95]; P < 0.001; I^2^ = 0%, 95%PI [−4.82, −1.37]) with low certainty of evidence compared with the IF alone. HOMA-IR was evaluated as an outcome measure in eight studies [42, 45, 47–51, 53], involving a total of 269 individuals. (Fig. 5). It was observed that the combined strategy showed lower HOMA-IR (SMD −0.57; 95%CI [−0.83, −0.31]; P < 0.001; I^2^ = 6%, 95%PI [−1.01, −0.13]) with low certainty of evidence compared with the IF alone.

### The Effects of IF + EX Versus IF Alone on Lipid Profiles

**Fig. 6:**
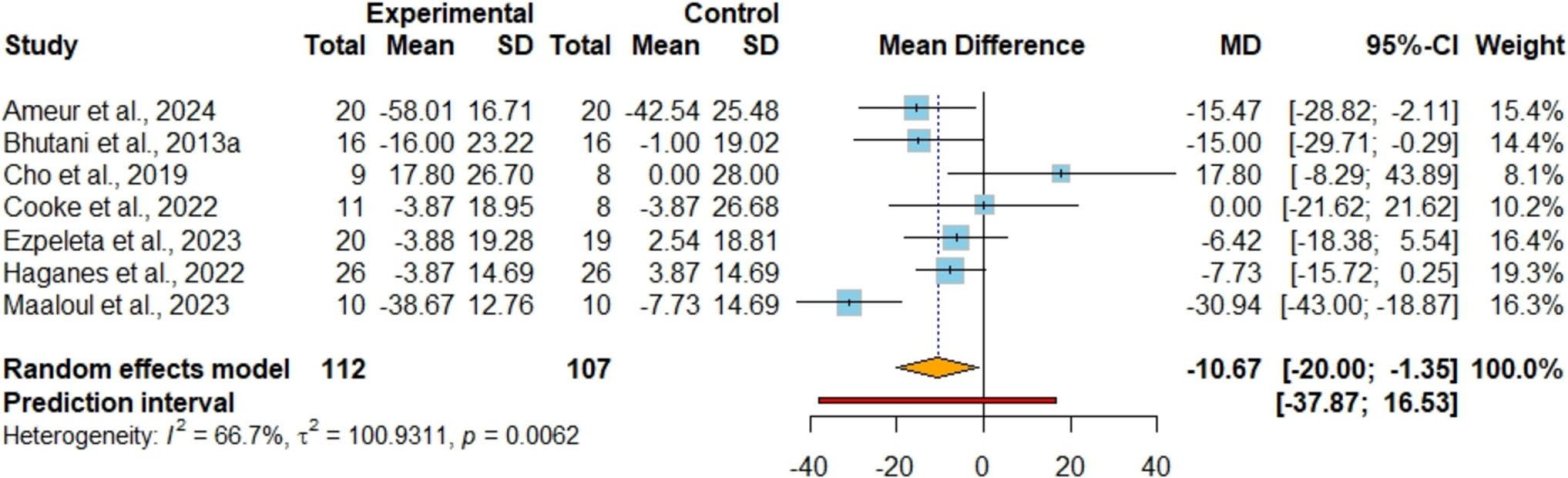
Meta-analysis of IF + EX vs. IF alone on low-density lipoprotein cholesterol. (MD)

Nine studies [42, 45, 47–53] were included in the TC analysis, with 290 participants evaluated (Figure S6). The analysis found no significant difference between the groups regarding TC levels (MD −7.39 mg/dl; 95%CI [−16.53, 1.74]; P = 0.11; I^2^ = 65%, 95%PI [−35.25, 20.46]), with evidence rated as having very low certainty. Sensitivity analyses demonstrated that removing the study by Ameur et al. [42] resolved heterogeneity, although it did not alter the statistical significance of the results. Eight studies [42, 45, 47–50, 52, 53], comprising 244 participants, evaluated HDL cholesterol as an outcome measure (Figure S7). The analysis showed no significant difference in HDL cholesterol levels between groups (MD 3.32 mg/dl; 95%CI [−1.56, 8.21]; P = 0.18; I^2^ = 81%, 95%PI [−12.72, 19.36]), with the certainty of evidence assessed as very low. After sensitivity analysis, we found that removing any study did not reduce heterogeneity. LDL cholesterol was analyzed in seven studies [42, 45, 47–50, 52], involving a total of 219 participants (Fig. 6). The results indicated the combined strategy showed a lower LDL level when compared to IF alone (MD −10.67 mg/dl; 95%CI [−20.00, −1.35]; P = 0.02; I^2^ = 67%, 95%PI [−37.87, 16.53]) with very low certainty of evidence. Sensitivity analysis demonstrated that excluding the study by Maaloul et al. [52] had no impact on statistical significance but lowered heterogeneity to 22%. Nine studies [42, 45, 47–53], including a total of 290 participants, evaluated TG levels as an outcome measure (Figure S8). The results showed no notable difference in TG levels between the groups (MD −12.86 mg/dl; 95%CI [−34.11, 8.40]; P = 0.24; I^2^ = 82%, 95%PI [−83.20, 57.48]), with the evidence quality assessed as very low. After sensitivity analysis, we found that removing any study did not reduce heterogeneity.

### The Effects of IF + EX Versus IF Alone on Blood Pressure and CRF

**Fig. 7:**
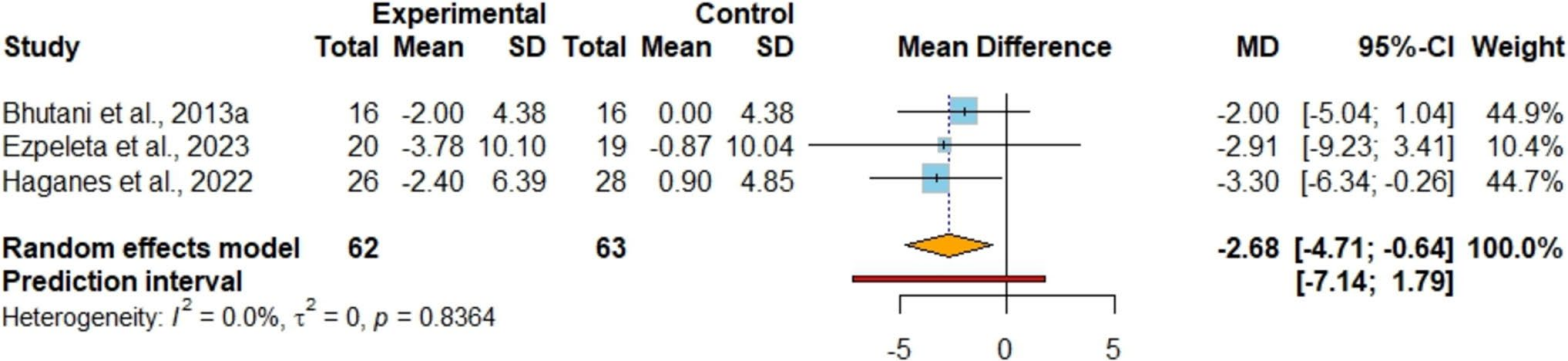
Meta-analysis of IF + EX vs. IF alone on resting heart rate. (MD)

**Fig. 8:**
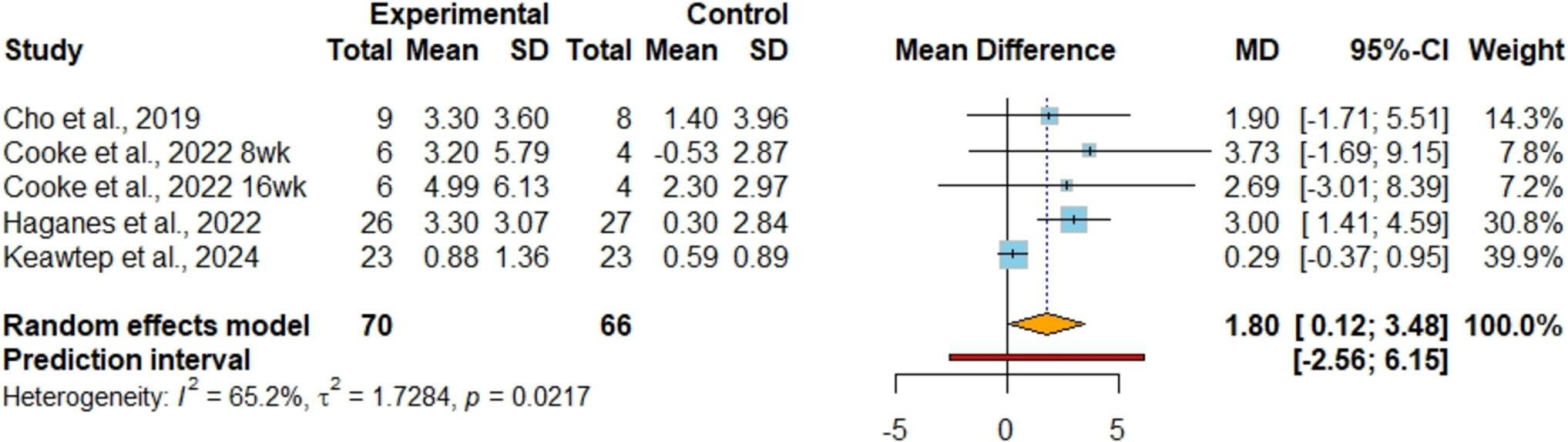
Meta-analysis of IF + EX vs. IF alone on VO_2 max_. (MD)

In the assessment of SBP and DBP across six studies [42, 45, 48–50, 53] involving 209 participants (Figure S9 and Figure S10), no significant differences were noted in SBP and DBP between the groups, supported by evidence of very low certainty. The mean differences were 0.16 mmHG (95%CI [−2.66, 2.99]; P = 0.91; I^2^ = 64%, 95%PI [−7.64, 7.97]) for SBP and −0.05 mmHg (95%CI [−1.98, 1.88]; P = 0.96; I^2^ = 53%, 95%PI [−5.10, 5.00]) for DBP. Sensitivity analyses indicated that removing the study by Ameur et al. [42] eliminated heterogeneity without altering the statistical significance of the results. In the analysis of resting heart rate from 3 studies [45, 49, 50] involving 125 participants (Fig. 7). It was observed that the combined strategy may result in a lower resting heart rate (MD −2.68 bpm; 95%CI [−4.71, −0.64]; P = 0.01; I^2^ = 0%, 95%PI [−7.14, 1.79]) with low certainty of evidence. VO_2 max_ was analyzed from 4 studies [47, 48, 50, 51], including a total of 136 participants (Fig. 8). It was observed that the combined strategy showed higher VO_2 max_ (MD 1.80 ml/kg/min; 95%CI [0.12, 3.48]; P = 0.036; I^2^ = 65%, 95%PI [−2.56, 6.15]) with very low certainty of evidence when compared to the IF alone. Upon performing sensitivity analysis, the exclusion of the trial conducted by Keawtep et al. [51] had no impact on the significance of the findings; however, it eliminated heterogeneity, reducing the I^2^ value to 0%.

### The Effects of IF + EX Versus IF Alone on Leptin, Adiponectin

In terms of leptin, the combined strategy showed a lower concentration (MD −13.76 ng/ml; 95%CI [−25.76, −1.76]; P = 0.02; I^2^ = 47%, 95%PI [−129.39, 101.87]) when compared to the IF alone with low certainty of evidence in 2 studies [46, 50] involving 82 participants (Figure S11). In the analysis of adiponectin levels from three studies [46, 50, 51] comprising 126 participants (Figure S12), the evidence indicated low certainty and showed no significant difference between the combined intervention and IF alone (SMD 0.35; 95%CI [0.00, 0.70]; P = 0.05; I^2^ = 0%, 95%PI [−0.42, 1.13]).

### Subgroup Analysis

Detailed results of the subgroup analysis are provided in Supplementary Table S2, which presents SMD/MD with corresponding 95% CIs for each subgroup separately. Significant group differences were observed in fasting glucose (based on the participant age and sex), LDL (based on the participant sex and IF type), TG (based on the participant age and sex, IF type and EX type) and VO_2 max_ (based on the participant age, intervention duration, and EX type), while the other outcomes did not observe any subgroup difference.

### Risk of Bias

The ROB 2 tool was used to assess the risk of bias in each study included. A summary of the overall assessments for all five domains of bias is presented in Table 2. Risk of bias assessment revealed that three studies [45, 46, 52] were categorized as high risk, whereas nine studies [42–44, 47–51, 53] presented some concerns. Among studies classified as having some concerns, all were missing details regarding deviations from intended interventions. Additionally, six studies [42–44, 47, 48, 51] did not adequately describe allocation concealment within the randomization process domain. Furthermore, one study [48] did not report the measurement of outcomes, and another study [42] lacked sufficient information concerning the selection of reported results. Among the studies with a high risk of bias, two [45, 46] had significant baseline differences between the intervention and control groups, together with randomization issues, and the third [52] lacked baseline information and a randomization process. The three studies identified as having a high risk of bias [45, 46, 52] also raised some concerns regarding the domains of deviations from the intended intervention, missing outcome data, and selection of the reported results. More specific assessment justification can be found in Table S3.

**Table 2:** Risk of bias assessment

Study	Randomization process	Deviations from the intended interventions	Missing outcome data	Measurement of the outcome	Selection of the reported result	Overall Bias
Ameur et al., 2024	Some concerns	Some concerns	Low	Low	Some concerns	Some concerns
Batitucci et al., 2022	Some concerns	Some concerns	Low	Low	Low	Some concerns
Batitucci et al., 2024	Some concerns	Some concerns	Low	Low	Low	Some concerns
Bhutani et al., 2013a	High	Some concerns	Low	Low	Some concerns	High
Bhutani et al., 2013b	High	Some concerns	Some concerns	Low	Some concerns	High
Cho et al., 2019	Some concerns	Some concerns	Low	Low	Low	Some concerns
Cooke et al., 2022	Some concerns	Some concerns	Low	Some concerns	Low	Some concerns
Ezpeleta et al., 2023	Low	Some concerns	Low	Low	Low	Some concerns
Haganes et al., 2022	Low	Some concerns	Low	Low	Low	Some concerns
Keawtep et al., 2024	Some concerns	Some concerns	Low	Low	Low	Some concerns
Maaloul et al., 2022	High	Some concerns	Low	Low	Some concerns	High
Oh et al., 2018	Low	Some concerns	Low	Low	Low	Some concerns

### Certainty of Evidence

The GRADE approach was utilized to evaluate the overall certainty of evidence, and the detailed assessment is summarized in Table S4. Among the 19 outcomes analyzed, the certainty of evidence was downgraded to moderate for fat mass. The certainty for BMI, WC, visceral fat, insulin, HOMA-IR, resting heart rate, leptin, and adiponectin were downgraded to low. Body mass, fat-free mass, fasting glucose, SBP, DBP, TC, TG, HDL, LDL, and VO_2 max_ were downgraded to very low certainty of evidence.

### Assessment of Study Quality and Reporting in Exercise

The quality of the included studies was evaluated using the TESTEX scale. A summary of the assessments across the twelve domains is provided in Table S5. Of the 12 included studies, nine were rated as good quality [42–47, 50, 52, 53], two as high quality [49, 51], and one as low quality [48].

### Publication Bias

We conducted a publication bias assessment to determine the potential impact of selective reporting on the findings of our meta-analysis. This assessment was specifically performed for body mass and fat mass outcomes, as these variables fulfilled the minimum criterion of having at least ten studies included (Table S6). Egger’s linear regression test was used to assess funnel plot asymmetry as an indicator of the risk of publication bias. The results indicated no significant risk of publication bias for fat mass (P = 0.06), but suggested a risk of publication bias for body mass (P = 0.0023).

## Discussion

This systematic review and meta-analysis investigated whether combining IF with exercise provides additional benefits on cardiometabolic health and body composition in adults with overweight/obesity. Our findings demonstrate significant improvements in CRF, body composition, and specific metabolic parameters when exercise is added to IF.

Previous systematic reviews and meta-analyses have consistently demonstrated weight loss following IF in adults with overweight/obesity [54]. However, this current study represents the first known investigation quantitatively assessing the additional effects of exercise on body composition in this population. The findings of this study indicate that incorporating exercise alongside IF resulted in significantly favorable effects on body composition rather than solely promoting weight loss. Specifically, significant reductions were observed in fat mass (P = 0.01), and WC (P < 0.001), while no significant changes were noted in body mass (P = 0.67), BMI (P = 0.21), and fat-free mass (P = 0.20). In our study, while total body mass reduction was comparable between the IF-only and combined intervention groups, the combined group exhibited significantly greater fat mass reduction. A recent network meta-analysis showed that combining ADF with moderate-intensity continuous training achieved the greatest weight and fat mass reductions, followed by ADF with exercise and the 5:2 diet with exercise among all IF and/or exercise interventions [32]. These findings indicate that while IF primarily drives overall weight loss, exercise plays a distinct role in optimizing body composition through targeted fat mass reduction. Prior research has highlighted the influences of training modalities during energy intake restriction on fat-mass loss [55], indicating that endurance exercise serves as a key predictor of fat-mass reduction in interventional studies. While resistance training during dietary restriction may not independently enhance fat-mass loss, it contributes to improved body composition through increases in fat-free mass. In our current meta-analysis, we examined various exercise modalities, including continuous training [45, 46, 49], concurrent training [47, 51–53], and high-intensity interval [43, 44, 48, 50]/functional [42] training. Despite conducting subgroup analyses, we observed no significant differences in weight management outcomes among exercise types when combined with IF, possibly due to the heterogeneity of existing protocols and limited available data. Therefore, well-designed RCTs are needed to elucidate the optimal combination of IF and specific exercise modalities. Further research is needed to establish optimal combinations of IF and exercise modalities for enhancing body composition and maintaining long-term weight management in individuals with overweight/obesity.

Various IF regimens have shown promise in reducing fasting insulin, insulin resistance, and HbA1c levels in healthy individuals with obesity and prediabetes [56]. IF diets also have certain therapeutic effects on blood glucose in patients with metabolic syndrome and significantly improve insulin resistance [14]. In adults with overweight/obesity, substantial evidence supports that exercise enhances glycemic control by boosting insulin sensitivity and glucose uptake in muscles [57]. Physical activity induces muscle contractions that prompt glucose transporters (GLUT4) to translocate to the cell surface, facilitating insulin-independent glucose entry [58]. Furthermore, exercise activates AMP-activated protein kinase (AMPK), a key enzyme that enhances insulin signaling and mitochondrial function, collectively improving the body's capacity to regulate blood sugar levels and reduce insulin resistance [59]. The findings of the meta-analysis indicate that incorporating exercise alongside IF could lead to reductions in HOMA-IR (−0.57 [95% CI: −0.83; −0.31], P = P < 0.001) and insulin levels (−3.1 uIU/ml [95% CI: −4.25; −1.95], P < 0.001). Although our meta-analysis showed no statistically significant differences in fasting glucose between the combined group versus IF alone, subgroup analyses revealed differential responses based on age and sex, highlighting the importance of considering these demographic factors when tailoring combined IF and exercise interventions. The combined intervention showed a larger reduction in LDL levels, consistent with evidence showing that exercise improves lipid profiles through LDL reduction and HDL increase [24]. Although our meta-analysis did not demonstrate statistically significant improvements in HDL, several individual studies reported greater HDL increases in the combined group [42, 45, 52, 53], the synergistic effect of combining IF with exercise appears to enhance lipid metabolism more effectively than IF alone, possibly through increased lipolysis and fat oxidation during fasting periods coupled with exercise-induced metabolic adaptations [60]. These findings suggest the need for larger-scale, well-designed RCTs to better elucidate the mechanisms underlying these potential beneficial effects on lipid metabolism in individuals with overweight/obesity.

CRF improvements were evident through increased VO_2 max_ and reduced resting heart rate in the combined group. Our qualitative synthesis indicated that the addition of exercise to IF leads to a lower resting heart rate (−2.68 bpm [95%CI: −4.71; −0.64], P = 0.01) and an elevation VO_2 max_ (1.80 ml/kg/min [95%CI: 0.12; 3.48], P = 0.036). While IF alone may maintain CRF in healthy adults [61], however, its impact on individuals with overweight/obesity remains ambiguous. Our findings indicate that exercise is necessary for enhancing this parameter during weight loss [62]. Exercise remains a key intervention for improving CRF, as supported by numerous meta-analyses across different populations and exercise types [63–65]. Low CRF levels are strongly associated with increased risk of cardiovascular disease, and all-cause mortality [28, 66]. Notably, higher CRF levels can help protect against obesity-related cardiovascular complications [62], highlighting the clinical significance of incorporating exercise into weight management programs.

No prior systematic review and meta-analysis have specifically evaluated the additional effect of incorporating exercise to IF on body composition and cardiometabolic health in adults with overweight/obesity. Our work highlights the relevance of combining exercise into IF intervention as an effective strategy to enhance body composition and cardiometabolic health in individuals with overweight/obesity. However, several limitations should be considered. The included studies used different exercise types and IF protocols, potentially affecting result interpretation. Furthermore, limiting our meta-analysis to English-language publications may have introduced bias by excluding relevant studies published in other languages. Also, it should be noted that the included studies are from around the globe, encompassing six continents and various ethnicities. The studies'relatively narrow age range limits applicability to middle-aged and older adults. Additionally, the limited number of available studies and the wide prediction intervals for some outcomes (fat mass, LDL, and cardiorespiratory fitness) necessitates further research to strengthen these findings. Future research should examine the effectiveness of different exercise modalities and fasting protocols across diverse populations. Large-scale RCTs are needed to identify optimal combinations of exercise modality and dietary approach for improving weight management and cardiometabolic health in individuals with overweight/obesity.

## Conclusion

In conclusion, integrating exercise into IF regimens may provide holistic health benefits beyond weight management alone, including improved glycemic control, and cardiovascular fitness. While these results are promising, more research is needed to confirm these findings and establish optimal intervention protocols. This meta-analysis provides valuable insights for healthcare practitioners in designing lifestyle interventions that combine both dietary and exercise strategies to optimize health outcomes in individuals with overweight/obesity.

## Key references

- Gabel K, Hamm A, Czyzewski O, Perez JS, Fought-Boudaia A, Motl RW, et al. A narrative review of intermittent fasting with exercise. Journal of the Academy of Nutrition and Dietetics. 2024.
  - This narrative review examines the combined effects of intermittent fasting (IF) and exercise, providing evidence that these interventions can synergistically reduce body weight and fat mass.
- Cheng X, Sun S, Chen M, Zhou X, Rao M, Guo D, et al. Evaluating the efficacy of intermittent fasting and exercise combinations for weight loss: A network meta‐analysis. Obesity Reviews.e13834.
  - This study is the first to apply network meta-analysis to compare various IF and exercise combinations, synthesizing data from relevant randomized controlled trials (RCTs). It identifies the most effective intervention strategies for weight loss, providing valuable evidence for optimizing IF and exercise regimens.
- Varady KA, Cienfuegos S, Ezpeleta M, Gabel K. Clinical application of intermittent fasting for weight loss: progress and future directions. Nature Reviews Endocrinology. 2022;18(5):309–21.
  - This review highlights the effects of intermittent fasting (IF) on body weight and cardiometabolic health, evaluates the safety of IF regimens, and offers practical guidance for incorporating IF into daily life.
- Lang JJ, Prince SA, Merucci K, Cadenas-Sanchez C, Chaput J-P, Fraser BJ, et al. Cardiorespiratory fitness is a strong and consistent predictor of morbidity and mortality among adults: an overview of meta-analyses representing over 20.9 million observations from 199 unique cohort studies. British journal of sports medicine. 2024;58(10):556–66.
  - This study highlights the strong predictive value of cardiorespiratory fitness for health outcomes and mortality, emphasizing its importance as a clinical risk stratification tool.
- Kazeminasab F, Baharlooie M, Karimi B, Mokhtari K, Rosenkranz SK, Santos HO. Effects of intermittent fasting combined with physical exercise on cardiometabolic outcomes: systematic review and meta-analysis of clinical studies. Nutrition Reviews. 2023:nuad155.
  - This meta-analysis finds that combining intermittent fasting with exercise leads to better improvements in weight loss, blood pressure, and lipid profiles compared to control diets with exercise.
- Ashcroft SP, Stocks B, Egan B, Zierath JR. Exercise induces tissue-specific adaptations to enhance cardiometabolic health. Cell metabolism. 2024.
  - This review explains how exercise-induced tissue-specific adaptations improve cardiometabolic health and highlights its benefits for preventing and treating non-communicable diseases.

## Supplementary Information

Below is the link to the electronic supplementary material.

Supplementary file1 (PDF 2768 KB)

## Data Availability

The datasets utilized and/or analyzed in this study can be ontained from the corresponding author upon reasonable request.
