# Supplementary material for: Additional Effect of Exercise to Intermittent Fasting on Body Composition and Cardiometabolic Health in Adults With Overweight/obesity: A Systematic Review and Meta-analysis
Source: Curr Obes Rep. 2025 Jun 19;14(1):54. doi: 10.1007/s13679-025-00645-9 (PMC12176969; doi:10.1007/s13679-025-00645-9)

Title: Additional effect of exercise to intermittent fasting on body composition and cardiometabolic health in adults with overweight/obesity: A systematic review and meta-analysis
First author: Zi-han Dai

Supplementary legend

Table S1. Search strategy
Table S2. Subgroup analysis results
Table S3. Risk of bias assessment justification
Table S4. Overall certainty of evidence
Table S5. Quality of the included studies
Table S6. Funnel plot for publication bias detection on body mass and fat mass

Figure S1. Meta-analysis of IF+EX vs. IF alone on body mass. Values are reported as mean difference (MD).
Figure S2. Meta-analysis of IF+EX vs. IF alone on body mass index. (MD)
Figure S3. Meta-analysis of IF+EX vs. IF alone on fat-free mass. (MD)
Figure S4. Meta-analysis of IF+EX vs. IF alone on visceral fat, presented as standardized mean differences (SMD).
Figure S5. Meta-analysis of IF+EX vs. IF alone on fasting glucose. (MD)
Figure S6. Meta-analysis of IF+EX vs. IF alone on total cholesterol. (MD)
Figure S7. Meta-analysis of IF+EX vs. IF alone on high-density lipoprotein cholesterol. (MD)
Figure S8. Meta-analysis of IF+EX vs. IF alone on triglycerides. (MD)
Figure S9. Meta-analysis of IF+EX vs. IF alone on Systolic blood pressure. (MD)
Figure S10. Meta-analysis of IF+EX vs. IF alone on diastolic blood pressure. (MD)
Figure S11. Meta-analysis of IF+EX vs. IF alone on leptin. (MD)
Figure S12. Meta-analysis of IF+EX vs. IF alone on adiponectin. (SMD)

Title: Additional effect of exercise to intermittent fasting on body composition and cardiometabolic health in adults with overweight/obesity: A systematic review and meta-analysis
First author: Zi-han Dai

Table S1. Search strategy

Database	Searches	Results
Embase	("Intermittent fasting" or "alternate-day fasting" or "intermittent energy restriction" or "intermittent calorie restriction" or "intermittent restrictive diet" or "periodic fasting" or "sporadic fasting" or "time-restricted feeding" or "time-restricted eating" or "5:2 diet" or "5:2 fasting" or "Ramadan" or "Ramadhan" or "time-restricted fasting" or "periodic diet" or "reduced meal frequency" or "alternate day modified fasting" or "modified alternate-day fasting" or "whole day fasting").mp. AND (Exercise* or aerobic* or running or jogging or walk* or hiking or swim* or aquatic* or cycling or bicycle* or strength* or physical activity* or fitness or train* or resistance).mp. AND (obesity or obese or overweight).mp.<br><br>Limiters - Full Text; Humans	769
Pubmed	(("Intermittent fasting"[Title/Abstract] OR "alternate-day fasting"[Title/Abstract] OR "intermittent energy restriction"[Title/Abstract] OR "periodic fasting"[Title/Abstract] OR "time-restricted feeding"[Title/Abstract] OR "time-restricted eating"[Title/Abstract] OR "5:2 diet"[Title/Abstract] OR "5:2 fasting"[Title/Abstract] OR "Ramadan"[Title/Abstract] OR "Ramadhan"[Title/Abstract] OR "time-restricted fasting"[Title/Abstract] OR "reduced meal frequency"[Title/Abstract] OR "alternate day modified fasting"[Title/Abstract] OR "modified alternate-day fasting"[Title/Abstract] OR "whole day fasting"[Title/Abstract]) AND (Exercise*[Title/Abstract] OR aerobic*[Title/Abstract] OR running[Title/Abstract] OR jogging[Title/Abstract] OR walk*[Title/Abstract] OR hiking[Title/Abstract] OR swim*[Title/Abstract] OR aquatic*[Title/Abstract] OR cycling[Title/Abstract] OR bicycle*[Title/Abstract] OR strength*[Title/Abstract] OR physical activity*[Title/Abstract] OR fitness[Title/Abstract] OR train*[Title/Abstract] OR resistance [Title/Abstract])) AND (obesity[Title/Abstract] OR obese[Title/Abstract] OR overweight[Title/Abstract])<br><br>Filters - Full text, Humans	333
SPORTDiscus	TX (("Intermittent fasting" or "alternate-day fasting" or "intermittent energy restriction" or "intermittent calorie restriction" or "intermittent restrictive diet" or "periodic fasting" or "sporadic fasting" or "time-restricted feeding" or "time-restricted eating" or "5:2 diet" or "5:2 fasting" or "Ramadan" or "Ramadhan" or "time-restricted fasting" or "periodic diet" or "reduced meal frequency" or "alternate day modified fasting" or "modified alternate-day fasting" or "whole day fasting") AND TX (Exercise* or aerobic* or running or jogging or walk* or hiking or swim* or aquatic* or cycling or bicycle* or strength* or physical activity* or fitness or train* or resistance) AND TX (obesity or obese or overweight)<br><br>Limiters - Full Text	465

Title: Additional effect of exercise to intermittent fasting on body composition and cardiometabolic health in adults with overweight/obesity: A systematic review and meta-analysis
First author: Zi-han Dai

Web of Science	((ALL=("Intermittent fasting" OR "alternate-day fasting" OR "intermittent energy restriction" OR "intermittent calorie restriction" OR "intermittent restrictive diet" OR "periodic fasting" OR "sporadic fasting" OR "time-restricted feeding" OR "time-restricted eating" OR "5:2 diet" OR "5:2 fasting" OR "Ramadan" OR "Ramadhan" OR "time-restricted fasting" OR "periodic diet" OR "reduced meal frequency" OR "alternate day modified fasting" OR "modified alternate-day fasting" OR "whole day fasting")) AND ALL=(Exercise* OR aerobic* OR running OR jogging OR walk* OR hiking OR swim* OR aquatic* OR cycling OR bicycle* OR strength* OR physical activity* OR fitness OR train* OR resistance)) AND ALL=(overweight or obese or obesity)<br><br>Limiters - N/A	736
Cochrane Library	"Intermittent fasting" or "alternate-day fasting" or "intermittent energy restriction" or "intermittent calorie restriction" or "intermittent restrictive diet" or "periodic fasting" or "sporadic fasting" or "time-restricted feeding" or "time-restricted eating" or "5:2 diet" or "5:2 fasting" or "Ramadan" or "Ramadhan" or "time-restricted fasting" or "periodic diet" or "reduced meal frequency" or "alternate day modified fasting" or "modified alternate-day fasting" or "whole day fasting" in All Text AND Overweight or obese or obesity in All Text AND Exercise* or aerobic* or running or jogging or walk* or hiking or swim* or aquatic* or cycling or bicycle* or strength* or physical activity* or fitness or train* or resistance in All Text<br><br>In Trials (Word variations have been searched)	331

Title: Additional effect of exercise to intermittent fasting on body composition and cardiometabolic health in adults with overweight/obesity: A systematic review and meta-analysis
First author: Zi-han Dai

Table S2. Subgroup analysis results

No. of studies		MD	95% CI	P	I^2	$P_{subgroup}$
**Body mass [kg]**						
Overall effect	10	-0.15	[-0.84; 0.54]	0.67	0%	
**Subgroup = age**						0.13
≤40 years	7	-0.76	[-1.88; 0.35]		0%	
>40 years	3	0.23	[-0.44; 0.89]		0%	
**Subgroup = duration**						0.15
≤8 weeks	4	-1.15	[-2.80; 0.49]		0%	
>8 weeks	6	0.12	[-0.48; 0.73]		0%	
**Subgroup = sex**						0.32
Female	4	-1.18	[-2.99; 0.62]		0%	
Male	1	-2.80	[-10.38; 4.78]		/	
Both	5	0.12	[-0.49; 0.72]		0%	
**Subgroup = EX type**						0.23
High-intensity interval training	4	-1.41	[-3.36; 0.54]		0%	
Continuous training	2	0.25	[-0.43; 0.93]		0%	
Concurrent exercise	4	-0.40	[-1.65; 0.84]		0%	
**Subgroup = IF type**						0.35
Time-restricted eating	2	-2.05	[-4.56; 0.47]		0%	
5:2 diet	3	-0.31	[-2.49; 1.87]		0%	
Alternate-day fasting	4	0.13	[-0.48; 0.74]		0%	
Ramadan intermittent fasting	1	-2.80	[-10.38; 4.78]		/	
**Body mass index [kg/m²]**						
Overall effect	7	-0.26	[-0.67; 0.15]	0.21	0%	
**Subgroup = age**						0.63
≤40 years	4	-0.33	[-0.84; 0.17]		0%	
>40 years	3	-0.12	[-0.82; 0.58]		0%	
**Subgroup = duration**						0.82
≤8 weeks	2	-0.33	[-1.08; 0.41]		0%	
>8 weeks	5	-0.23	[-0.72; 0.27]		0%	
**Subgroup = sex**						0.68
Female	3	-0.39	[-1.13; 0.35]		0%	
Both	4	-0.20	[-0.70; 0.29]		0%	
**Subgroup = EX type**						0.82
High-intensity interval training	2	-0.55	[-1.47; 0.36]		0%	
Continuous training	2	-0.24	[-1.44; 0.95]		44.2%	
Concurrent exercise	3	-0.21	[-0.76; 0.34]		0%	
**Subgroup = IF type**						0.59
Time-restricted eating	1	-0.90	[-2.19; 0.39]		/	
5:2 diet	2	-0.14	[-1.04; 0.76]		0%	
Alternate-day fasting	4	-0.20	[-0.70; 0.29]		0%	

Title: Additional effect of exercise to intermittent fasting on body composition and cardiometabolic health in adults with overweight/obesity: A systematic review and meta-analysis
First author: Zi-han Dai

No. of studies		MD	95% CI	P	I^2	$P_{subgroup}$
Fat mass [kg]						
Overall effect	10	-0.93	[-1.69; -0.18]	0.01	27%	
**Subgroup = age**						0.94
≤40 years	7	-0.97	[-1.87; -0.06]		30%	
>40 years	3	-1.04	[-2.77; 0.68]		47%	
**Subgroup = duration**						0.93
≤8 weeks	4	-1.12	[-2.19; -0.05]		0%	
>8 weeks	6	-1.04	[-2.45; 0.36]		57%	
**Subgroup = sex**						0.26
Female	4	-1.61	[-2.90; -0.33]		30%	
Male	1	-2.20	[-6.36; 1.96]		/	
Both	5	-0.44	[-1.26; 0.38]		24%	
**Subgroup = EX type**						0.80
High-intensity interval training	4	-1.35	[-3.14; 0.44]		53%	
Continuous exercise	2	-1.23	[-4.22; 1.75]		73%	
Concurrent exercise	4	-0.70	[-1.70; 0.30]		0%	
**Subgroup = IF type**						0.54
Time-restricted eating	2	-2.66	[-5.70; 0.37]		61%	
5:2 diet	3	-0.61	[-1.89; 0.67]		0%	
Alternate-day fasting	4	-0.59	[-1.52; 0.35]		34%	
Ramadan intermittent fasting	1	-2.20	[-6.36; 1.96]		/	
Fat free mass [kg]						
Overall effect	9	0.75	[-0.41; 1.90]	0.20	86%	
**Subgroup = age**						0.54
≤40 years	6	0.88	[-0.86; 2.61]		91%	
>40 years	3	0.30	[-0.37; 0.98]		0%	
**Subgroup = duration**						0.30
≤8 weeks	4	0.05	[-0.39; 0.50]		0%	
>8 weeks	5	1.12	[-0.84; 3.08]		91%	
**Subgroup = sex**						0.58
Female	4	1.33	[-0.87; 3.54]		94%	
Male	1	0.40	[-3.64; 4.44]		/	
Both	4	0.14	[-0.34; 0.62]		0%	
**Subgroup = EX type**						0.64
High-intensity interval training	4	1.13	[-1.35; 3.62]		94%	
Continuous exercise	2	-0.48	[-0.53; 1.43]		0%	
Concurrent exercise	3	-0.21	[-0.36; 0.60]		0%	
**Subgroup = IF type**						0.87
Time-restricted eating	2	2.18	[-2.52; 6.89]		98%	
5:2 diet	3	0.17	[-0.62; 0.95]		0%	
Alternate-day fasting	3	0.19	[-0.30; 0.68]		0%	
Ramadan intermittent fasting	1	0.40	[-3.64; 4.44]		/	

Title: Additional effect of exercise to intermittent fasting on body composition and cardiometabolic health in adults with overweight/obesity: A systematic review and meta-analysis
First author: Zi-han Dai

No. of studies		MD/SMD	95% CI	P	I^2	$P_{subgroup}$
Waist circumference [cm]						
Overall effect	7	-2.51	[-3.70; -1.32]	<0.001	0%	
**Subgroup = age**						0.33
≤40 years	5	-3.12	[-4.66; -1.58]		0%	
>40 years	2	-1.66	[-4.17; 0.86]		44%	
**Subgroup = duration**						0.96
≤8 weeks	3	-2.58	[-4.46; -0.70]		0%	
>8 weeks	4	-2.64	[-4.64; -0.65]		35%	
**Subgroup = sex**						0.56
Female	2	-3.37	[-5.39; -1.35]		0%	
Male	1	-3.20	[-8.43; 2.03]		/	
Both	4	-1.98	[-3.59; -0.38]		15%	
**Subgroup = EX type**						0.43
High-intensity interval training	3	-3.62	[-5.54; -1.70]		0%	
Continuous exercise	2	-1.66	[-4.17; 0.86]		44%	
Concurrent exercise	2	-2.21	[-4.78; 0.36]		0%	
**Subgroup = IF type**						0.31
Time-restricted eating	2	-3.65	[-6.23; -1.08]		0%	
5:2 diet	2	-3.49	[-6.01; -0.97]		0%	
Alternate-day fasting	3	-1.70	[-3.28; -0.11]		0%	
Visceral fat mass						
Overall effect	4	-0.20	[-0.55; 0.15]	0.26	6%	
**Subgroup = age**						0.38
≤40 years	3	-0.30	[-0.72; 0.11]		17%	
>40 years	1	0.03	[-0.59; 0.66]		/	
**Subgroup = duration**						0.81
≤8 weeks	1	-0.27	[-0.80; 0.25]		/	
>8 weeks	3	-0.18	[-0.74; 0.38]		35%	
**Subgroup = sex**						0.81
Female	1	-0.27	[-0.80; 0.25]		/	
Both	3	-0.18	[-0.74; 0.38]		35%	
**Subgroup = EX type**						0.27
High-intensity interval training	2	-0.17	[-0.63; 0.28]		0%	
Continuous exercise	1	0.03	[-0.59; 0.66]		/	
Concurrent exercise	1	-0.95	[-1.97; 0.07]		/	
**Subgroup = IF type**						0.71
Time-restricted eating	1	-0.27	[-0.80; 0.25]		/	
5:2 diet	1	0.12	[-0.79; 1.04]		/	
Alternate-day fasting	2	-0.37	[-1.33; 0.58]		62%	
Fasting glucose [mg/dl]						
Overall effect	9	-1.92	[-4.47; 0.62]	0.14	51%	
**Subgroup = age**						0.02

Title: Additional effect of exercise to intermittent fasting on body composition and cardiometabolic health in adults with overweight/obesity: A systematic review and meta-analysis
First author: Zi-han Dai

No. of studies		MD/SMD	95% CI	P	I^2	$P_{subgroup}$
≤40 years	6	-3.48	[-6.52; -0.44]		53%	
>40 years	3	1.71	[-1.16; 4.57]		0%	
**Subgroup = duration**						0.29
≤8 weeks	3	-4.10	[-9.74; 1.55]		79%	
>8 weeks	6	-0.74	[-3.49; 2.02]		15%	
**Subgroup = sex**						**0.04**
Female	3	-0.31	[-3.53; 2.92]		57%	
Male	1	-7.21	[-11.54; -2.87]		/	
Both	5	-1.65	[-4.81; 1.50]		0%	
**Subgroup = EX type**						0.56
High-intensity interval training	3	-1.37	[-4.01; 1.27]		27%	
Continuous exercise	2	0.64	[-4.55; 5.82]		0%	
Concurrent exercise	4	-3.58	[-9.18; 2.03]		76%	
**Subgroup = IF type**						0.07
Time-restricted eating	2	-1.79	[-6.39; 2.81]		62%	
5:2 diet	2	0.52	[-3.33; 4.37]		44%	
Alternate-day fasting	4	-1.53	[-5.80; 2.73]		0%	
Ramadan intermittent fasting	1	-7.21	[-11.54; -2.87]		/	
**Insulin (uIU/ml)**						
Overall effect	7	-3.10	[-4.25; -1.95]	<0.001	0%	
**Subgroup = age**						0.76
≤40 years	4	-3.30	[-5.18; -1.41]		24%	
>40 years	3	-2.87	[-4.82; -0.91]		0%	
**Subgroup = duration**						0.37
≤8 weeks	2	-2.23	[-4.80; 0.35]		12%	
>8 weeks	5	-3.54	[-4.82; -2.27]		0%	
**Subgroup = sex**						0.55
Female	3	-2.98	[-4.26; -1.69]		22%	
Both	4	-4.23	[-8.17; -0.30]		0%	
**Subgroup = EX type**						0.83
High-intensity interval training	2	-2.89	[-5.05; -0.73]		61%	
Continuous exercise	2	-1.63	[-7.69; 4.43]		0%	
Concurrent exercise	3	-3.44	[-5.35; -1.52]		0%	
**Subgroup = IF type**						0.83
Time-restricted eating	2	-2.89	[-5.05; -0.73]		61%	
5:2 diet	1	-3.01	[-5.07; -0.95]		/	
Alternate-day fasting	4	-4.23	[-8.17; -0.30]		0%	
**HOMA-IR**						
Overall effect	8	-0.57	[-0.83; -0.31]	<0.001	6%	
**Subgroup = age**						0.17
≤40 years	5	-0.74	[-1.07; -0.41]		0%	
>40 years	3	-0.35	[-0.79; 0.08]		28%	

Title: Additional effect of exercise to intermittent fasting on body composition and cardiometabolic health in adults with overweight/obesity: A systematic review and meta-analysis
First author: Zi-han Dai

No. of studies		MD/SMD	95% CI	P	I^2	**$P_{subgroup}$**
**Subgroup = duration**						0.71
≤8 weeks	2	-0.65	[-1.11; -0.19]		0%	
>8 weeks	6	-0.54	[-0.90; -0.17]		32%	
**Subgroup = sex**						0.05
Female	3	-0.81	[-1.16; -0.46]		0%	
Both	5	-0.33	[-0.68; 0.02]		0%	
**Subgroup = EX type**						0.10
High-intensity interval training	3	-0.75	[-1.16; -0.33]		17%	
Continuous exercise	2	-0.13	[-0.60; 0.34]		0%	
Concurrent exercise	3	-0.73	[-1.17; -0.30]		0%	
**Subgroup = IF type**						0.33
Time-restricted eating	2	-0.56	[-1.15; 0.02]		79%	
5:2 diet	2	-0.35	[-0.92; 0.22]		73%	
Alternate-day fasting	4	-1.26	[-2.29; -0.22]		0%	
Low-density lipoprotein [mg/dl]						
Overall effect	7	-10.67	[-20.00; -1.35]	0.02	67%	
**Subgroup = age**						0.99
≤40 years	5	-9.72	[-24.11; 4.66]		76%	
>40 years	2	-9.83	[-19.11; -0.56]		0%	
**Subgroup = duration**						0.37
≤8 weeks	2	-18.88	[-41.60; 3.84]		90%	
>8 weeks	5	-7.98	[-15.78; -0.18]		38%	
**Subgroup = sex**						**<0.01**
Female	2	-9.77	[-16.63; -2.92]		0%	
Male	1	-30.94	[-43.00; -18.87]		/	
Both	4	-4.73	[-15.05; 5.60]		39%	
**Subgroup = EX type**						0.99
High-intensity interval training	3	-8.88	[-15.41; -2.34]		0%	
Continuous exercise	2	-9.83	[-19.11; -0.56]		0%	
Concurrent exercise	2	-8.00	[-55.68; 39.68]		91%	
**Subgroup = IF type**						**<0.01**
Time-restricted eating	2	-9.77	[-16.63; -2.92]		0%	
5:2 diet	1	0.00	[-21.62; 21.62]		/	
Alternate-day fasting	3	-4.52	[-19.50; 10.46]		57%	
Ramadan intermittent fasting	1	-30.94	[-43.00; -18.87]		/	
High-density lipoprotein [mg/dl]						
Overall effect	8	3.32	[-1.56; 8.21]	0.18	81%	
**Subgroup = age**						0.91
≤40 years	6	3.56	[-2.37; 9.49]		81%	
>40 years	2	2.77	[-9.48; 15.01]		90%	
**Subgroup = duration**						0.28
≤8 weeks	3	0.36	[-4.42; 5.15]		75%	

Title: Additional effect of exercise to intermittent fasting on body composition and cardiometabolic health in adults with overweight/obesity: A systematic review and meta-analysis
First author: Zi-han Dai

No. of studies		MD/SMD	95% CI	P	I^2	$P_{subgroup}$
>8 weeks	5	5.37	[-2.29; 13.02]		83%	
**Subgroup = sex**						0.90
Female	2	7.39	[-15.34; 30.12]		96%	
Male	1	3.09	[-0.63; 6.82]		/	
Both	5	2.36	[-2.22; 6.93]		63%	
**Subgroup = EX type**						0.97
High-intensity interval training	3	4.80	[-9.09; 18.69]		92%	
Continuous exercise	2	2.77	[-9.48; 15.01]		90%	
Concurrent exercise	3	3.01	[0.02; 6.00]		0%	
**Subgroup = IF type**						0.88
Time-restricted eating	2	7.39	[-15.34; 30.12]		96%	
5:2 diet	1	0.00	[-7.87; 7.87]		/	
Alternate-day fasting	4	2.83	[-2.64; 8.30]		71%	
Ramadan intermittent fasting	1	3.09	[-0.63; 6.82]		/	
**Total cholesterol [mg/dl]**						
Overall effect	9	-7.39	[-16.53; 1.74]	0.11	65%	
**Subgroup = age**						0.75
≤40 years	6	-7.87	[-21.83; 6.09]		76%	
>40 years	3	-5.21	[-13.56; 3.13]		12%	
**Subgroup = duration**						0.50
≤8 weeks	3	-3.60	[-16.12; 8.91]		61%	
>8 weeks	6	-9.70	[-22.53; 3.13]		69%	
**Subgroup = sex**						0.30
Female	3	-12.19	[-33.34; 8.97]		86%	
Male	1	-15.47	[-30.14; -0.79]		/	
Both	5	-2.06	[-12.42; 8.30]		29%	
**Subgroup = EX type**						0.38
High-intensity interval training	3	-15.77	[-36.60; 5.05]		84%	
Continuous exercise	2	-8.81	[-20.24; 2.62]		15%	
Concurrent exercise	4	-0.13	[-12.37; 12.11]		51%	
**Subgroup = IF type**						0.32
Time-restricted eating	2	-18.64	[-48.92; 11.65]		92%	
5:2 diet	2	-0.84	[-13.34; 11.67]		0%	
Alternate-day fasting	4	-1.27	[-13.19; 10.65]		46%	
Ramadan intermittent fasting	1	-15.47	[-30.14; -0.79]		/	
**Triglycerides [mg/dl]**						
Overall effect	9	-12.86	[-34.11; 8.40]	0.24	82%	
**Subgroup = age**						**0.04**
≤40 years	6	-26.09	[-55.03; 2.84]		87%	
>40 years	3	5.77	[-4.29; 15.83]		17%	
**Subgroup = duration**						0.31
≤8 weeks	3	-31.27	[-83.59; 21.04]		94%	

Title: Additional effect of exercise to intermittent fasting on body composition and cardiometabolic health in adults with overweight/obesity: A systematic review and meta-analysis
First author: Zi-han Dai

No. of studies		MD/SMD	95% CI	P	I^2	$P_{subgroup}$
>8 weeks	6	-3.06	[-19.51; 13.39]		59%	
**Subgroup = sex**						**<0.01**
Female	3	-4.49	[-32.94; 23.97]		85%	
Male	1	-62.00	[-91.32; -32.68]		/	
Both	5	-7.66	[-37.02; 21.70]		69%	
**Subgroup = EX type**						**0.03**
High-intensity interval training	3	-3.29	[-31.85; 25.28]		85%	
Continuous exercise	2	11.37	[-8.81; 31.55]		42%	
Concurrent exercise	4	-40.65	[-74.01; -7.29]		72%	
**Subgroup = IF type**						**<0.01**
Time-restricted eating	2	-5.65	[-53.35; 42.05]		92%	
5:2 diet	2	-2.00	[-19.75; 15.75]		0%	
Alternate-day fasting	4	-12.22	[-53.40; 28.96]		77%	
Ramadan intermittent fasting	1	-62.00	[-91.32; -32.68]		/	
**VO_{2max} [ml/kg/min]**						
Overall effect	5	1.80	[0.12; 3.48]	0.036	65%	
**Subgroup = age**						**<0.01**
≤40 years	4	2.87	[1.50; 4.24]		0%	
>40 years	1	0.29	[-0.37; 0.95]		/	
**Subgroup = duration**						**<0.01**
≤8 weeks	2	3.06	[1.53; 4.59]		0%	
>8 weeks	3	0.38	[-0.29; 1.05]		0%	
**Subgroup = sex**						0.61
Female	2	1.54	[-1.10; 4.19]		89%	
Both	3	2.51	[-0.15; 5.17]		0%	
**Subgroup = EX type**						**<0.01**
High-intensity interval training	3	3.03	[1.56; 4.51]		0%	
Concurrent exercise	2	0.34	[-0.31; 1.00]		0%	
**Subgroup = IF type**						0.32
Time-restricted eating	1	3.00	[1.41; 4.59]		/	
5:2 diet	3	0.99	[-1.08; 3.07]		7.7%	
Alternate-day fasting	1	1.90	[-1.71; 5.51]		/	
**Resting heart rate [rpm]**						
Overall effect	3	-2.68	[-4.71; -0.64]	0.01	0%	
**Subgroup = age**						0.59
≤40 years	1	-3.30	[-6.34; -0.26]		/	
>40 years	2	-2.17	[-4.91; 0.57]		0%	
**Subgroup = duration**						0.59
≤8 weeks	1	-3.30	[-6.34; -0.26]		/	
>8 weeks	2	-2.17	[-4.91; 0.57]		0%	
**Subgroup = sex**						0.59
Female	1	-3.30	[-6.34; -0.26]		/	

Title: Additional effect of exercise to intermittent fasting on body composition and cardiometabolic health in adults with overweight/obesity: A systematic review and meta-analysis
First author: Zi-han Dai

No. of studies		MD/SMD	95% CI	P	I^2	$P_{subgroup}$
Both	2	-2.17	[-4.91; 0.57]		0%	
**Subgroup = EX type**						0.59
High-intensity interval training	1	-3.30	[-6.34; -0.26]		/	
Continuous exercise	2	-2.17	[-4.91; 0.57]		0%	
**Subgroup = IF type**						0.59
Time-restricted eating	1	-3.30	[-6.34; -0.26]		/	
Alternate-day fasting	2	-2.17	[-4.91; 0.57]		0%	
Systolic blood pressure [mmHg]						
Overall effect	6	0.16	[-2.66; 2.99]	0.91	64%	
**Subgroup = age**						0.55
≤40 years	4	-0.22	[-4.05; 3.62]		77%	
>40 years	2	1.43	[-2.39; 5.24]		0%	
**Subgroup = duration**						0.62
≤8 weeks	2	1.21	[-2.46; 3.77]		31%	
>8 weeks	4	-0.16	[-4.10; 3.77]		71%	
**Subgroup = sex**						0.52
Female	2	-1.11	[-8.17; 5.95]		90%	
Both	4	1.35	[-1.23; 3.93]		0%	
**Subgroup = EX type**						0.70
High-intensity interval training	3	0.14	[-4.83; 5.11]		84%	
Continuous exercise	2	1.43	[-2.39; 5.24]		0%	
Concurrent exercise	1	-1.50	[-7.16; 4.16]		/	
**Subgroup = IF type**						0.55
Time-restricted eating	2	-1.11	[-8.17; 5.95]		90%	
5:2 diet	1	3.00	[-1.44; 7.44]		/	
Alternate-day fasting	3	0.51	[-2.65; 3.68]		0%	
Diastolic blood pressure [mmHg]						
Overall effect	6	-0.05	[-1.98; 1.88]	0.96	53%	
**Subgroup = age**						0.90
≤40 years	4	-0.17	[-2.61; 2.28]		60%	
>40 years	2	0.14	[-4.14; 4.43]		59%	
**Subgroup = duration**						0.97
≤8 weeks	2	-0.02	[-2.39; 2.36]		0%	
>8 weeks	4	0.04	[-2.80; 2.89]		71%	
**Subgroup = sex**						0.21
Female	2	-1.24	[-3.77; 1.30]		57%	
Both	4	0.98	[-1.35; 3.31]		30%	
**Subgroup = EX type**						0.95
High-intensity interval training	3	0.02	[-2.98; 3.03]		73%	
Continuous exercise	2	0.14	[-4.14; 4.43]		59%	
Concurrent exercise	1	-0.90	[-6.25; 4.45]		/	
**Subgroup = IF type**						0.15

Title: Additional effect of exercise to intermittent fasting on body composition and cardiometabolic health in adults with overweight/obesity: A systematic review and meta-analysis
First author: Zi-han Dai

No. of studies		MD/SMD	95% CI	P	I^2	**$P_{subgroup}$**
Time-restricted eating	2	-1.24	[-3.77; 1.30]		57%	
5:2 diet	1	3.00	[-0.43; 6.43]		/	
Alternate-day fasting	3	0.05	[-2.90; 3.00]		27%	
Leptin [ng/ml]						
Overall effect	2	-13.76	[-25.76; -1.76]	0.02	47%	
**Subgroup = age**						0.17
≤40 years	1	-10.10	[-14.71; -5.49]		/	
>40 years	1	-24.00	[-43.30; -4.70]		/	
**Subgroup = duration**						0.17
≤8 weeks	1	-10.10	[-14.71; -5.49]		/	
>8 weeks	1	-24.00	[-43.30; -4.70]		/	
**Subgroup = sex**						0.17
Female	1	-10.10	[-14.71; -5.49]		/	
Both	1	-24.00	[-43.30; -4.70]		/	
**Subgroup = EX type**						0.17
High-intensity interval training	1	-10.10	[-14.71; -5.49]		/	
Continuous exercise	1	-24.00	[-43.30; -4.70]		/	
**Subgroup = IF type**						0.17
Time-restricted eating	1	-10.10	[-14.71; -5.49]		/	
Alternate-day fasting	1	-24.00	[-43.30; -4.70]		/	
Adiponectin						
Overall effect	3	0.35	[0.00; 0.70]	0.05	0%	
**Subgroup = age**						0.34
≤40 years	1	0.14	[-0.43; 0.70]		/	
>40 years	2	0.49	[0.04; 0.94]		0%	
**Subgroup = duration**						0.34
≤8 weeks	1	0.14	[-0.43; 0.70]		/	
>8 weeks	2	0.49	[0.04; 0.94]		0%	
**Subgroup = sex**						0.57
Female	2	0.29	[-0.12; 0.70]		0%	
Both	1	0.53	[-0.18; 1.24]		/	
**Subgroup = EX type**						0.63
High-intensity interval training	1	0.14	[-0.43; 0.70]		/	
Continuous exercise	1	0.53	[-0.18; 1.24]		/	
Concurrent exercise	1	0.46	[-0.13; 1.04]		/	
**Subgroup = IF type**						0.63
Time-restricted eating	1	0.14	[-0.43; 0.70]		/	
5:2 diet	1	0.46	[-0.13; 1.04]		/	
Alternate-day fasting	1	0.53	[-0.18; 1.24]		/	

Note: CI, confidence interval; EX, exercise; HOMA-IR, homeostatic model assessment for insulin resistance; IF, intermittent fasting; MD, mean difference; SMD, standardized mean difference; VO_{2max}, maximal oxygen uptake

Title: Additional effect of exercise to intermittent fasting on body composition and cardiometabolic health in adults with overweight/obesity: A systematic review and meta-analysis
First author: Zi-han Dai

Table S3. Risk of bias assessment justification

Ameur et al., 2024

Risk of bias		
Bias	Authors' judgment	Support for judgment
Randomization process	Some concerns	Quote: "The randomization of the participants was carried out using a web-based system for random number generation by a person independent of the research group."<br>There is no information regarding if the allocation sequence was concealed until participants were enrolled and assigned to interventions. And the baseline differences may suggest a problem with the randomization process.
Deviations from the intended interventions	Some concerns	No information about the blinding process of participants and carers. No information about deviations from the intervention.
Missing outcome data	Low	Report data from all randomized participants.
Measurement of the outcome	Low	For the body composition outcomes and biomarker outcomes, the measurement using standardized measures is relatively robust. Although theoretically, the recorded measures could be influenced by knowledge of the intervention, this is highly unlikely.
Selection of the reported result	Some concerns	Although results were reported in line with the article method part, the proposed data analysis cannot be found.
Overall Bias	Some concerns	Some concerns in the randomization process, the deviations from the intended interventions, and the selection of the reported result, but no issues about any of the other domains assessed.

Title: Additional effect of exercise to intermittent fasting on body composition and cardiometabolic health in adults with overweight/obesity: A systematic review and meta-analysis
First author: Zi-han Dai

Batitucci et al., 2022

Risk of bias		
Bias	Authors' judgment	Support for judgment
Randomization process	Some concerns	Quote: "A random draw was performed and the participants were divided into three groups"; "It can be observed that for some anthropometric parameters, lipid profile, and fasting glucose, the groups showed differences prior to the beginning of the interventions." There are no issues with the randomization process; however, the baseline differences may suggest a problem with the randomization process.
Deviations from the intended interventions	Some concerns	No information about the blinding process of participants and carers. No information about deviations from the intervention.
Missing outcome data	Low	Report data from all randomized participants.
Measurement of the outcome	Low	Quote: "All of the evaluators for physical tests remain blind to the treatments". For the other body composition outcomes and biomarker outcomes, the measurement using standardized measures is relatively robust. Although theoretically, the recorded measures could be influenced by knowledge of the intervention, this is highly unlikely.
Selection of the reported result	Low	Results were reported in line with the proposed data analysis outlined in the article, and all data reported to be collected was reported in full.
Overall Bias	Some concerns	Some concerns in the randomization process and the deviations from the intended interventions, but no issues in relation to any of the other domains assessed.

Title: Additional effect of exercise to intermittent fasting on body composition and cardiometabolic health in adults with overweight/obesity: A systematic review and meta-analysis
First author: Zi-han Dai

Batitucci et al., 2024

Risk of bias		
Bias	Authors' judgment	Support for judgment
Randomization process	Some concerns	Quote: "After a careful eligibility process, a randomized clinical trial was carried out which included 60 women with obesity, sedentary and without comorbidities, randomly (drawn) divided into 3 groups."<br>There are no issues with the randomization process; however, no information regarding the baseline differences between intervention groups.
Deviations from the intended interventions	Some concerns	No information about the blinding process of participants and carers. No information about deviations from the intervention.
Missing outcome data	Low	Report data from all randomized participants.
Measurement of the outcome	Low	The use of standardized measures is relatively robust for microbiota composition and other biomarker outcomes. Although theoretically, the recorded measures could be influenced by knowledge of the intervention, this is highly unlikely.
Selection of the reported result	Low	Results were reported in line with the proposed data analysis outlined in the article, and all data reported to be collected was reported in full.
Overall Bias	Some concerns	Some concerns in the randomization process and the deviations from the intended interventions, but no issues in relation to any of the other domains assessed.

Title: Additional effect of exercise to intermittent fasting on body composition and cardiometabolic health in adults with overweight/obesity: A systematic review and meta-analysis
First author: Zi-han Dai

Bhutani et al., 2013a

Risk of bias		
Bias	Authors' judgment	Support for judgment
Randomization process	High	Quote: "Subjects were recruited and randomized by the clinical coordinator (SB). Randomization was performed for each stratum by selecting an intervention at random from an opaque envelope."; "There were no between-group differences for age, sex, ethnicity, body weight, height, BMI, waist circumference, plasma lipids, or heart rate at baseline. Systolic and diastolic blood pressure values, however, differed between groups at baseline."; "Our randomization procedure may be flawed in that we chose to randomize additional subjects into groups that had high dropout rates. This uneven allocation of subjects to the intervention groups may have negatively impacted the internal validity of the study." There may exist issues with the randomization process. In addition, the baseline differences may suggest a problem with the randomization process.
Deviations from the intended interventions	Some concerns	There is no information on the blinding process of participants and carers. No information about deviations from the intervention.
Missing outcome data	Low	Quote: "Characteristics of the dropouts were not significantly different from those of the completers."
Measurement of the outcome	Low	For the body composition outcomes and biomarker outcomes, the measurement using standardized measures is relatively robust. Although theoretically, the recorded measures could be influenced by knowledge of the intervention, this is highly unlikely.
Selection of the reported result	Some concerns	Although results were reported in line with the article method part, the proposed data analysis cannot be found.
Overall Bias	High	Some concerns about the deviations from the intended interventions, and selection of the reported result; as high bias regarding the randomization process, while no issues about the other domains assessed.

Title: Additional effect of exercise to intermittent fasting on body composition and cardiometabolic health in adults with overweight/obesity: A systematic review and meta-analysis
First author: Zi-han Dai

Bhutani et al., 2013b

Risk of bias		
Bias	Authors' judgment	Support for judgment
Randomization process	High	Quote: "Randomization was performed for each stratum by selecting an intervention at random from an opaque envelope."; "There were no between-group differences for age, sex, body weight, fat mass, waist circumference, and adipokines. Systolic and diastolic blood pressures were higher in the ADF group at baseline versus the other groups."; "Additional subjects were randomized to groups that had high dropout rates (i.e. the ADF and exercise group) to ensure that the total number of subjects would be the same in each group at the end of the study."<br>There may exist issues with the randomization process. In addition, the baseline differences may suggest a problem with the randomization process.
Deviations from the intended interventions	Some concerns	There is no information on the blinding process of participants and carers. No information about deviations from the intervention.
Missing outcome data	Some concerns	No information about the missing data
Measurement of the outcome	Low	For the body composition outcomes and biomarker outcomes, the measurement using standardized measures is relatively robust. Although theoretically, the recorded measures could be influenced by knowledge of the intervention, this is highly unlikely.
Selection of the reported result	Some concerns	Although results were reported in line with the article method part, the proposed data analysis cannot be found.
Overall Bias	High	Some concerns about the deviations from the intended interventions, missing outcome data and selection of the reported result; as high bias regarding the randomization process, while no issues about the other domains assessed.

Title: Additional effect of exercise to intermittent fasting on body composition and cardiometabolic health in adults with overweight/obesity: A systematic review and meta-analysis
First author: Zi-han Dai

Cho et al., 2019

Risk of bias		
Bias	Authors' judgment	Support for judgment
Randomization process	Some concerns	Quote: "We randomly assigned participants in a 1:1:1:1 ratio to four groups. Block randomization was performed with a computer-generated random number sequence. An independent statistician generated the allocation sequence, and the study coordinator assigned the participants to interventions in chronological order as the participants enrolled. Only outcome assessors were blinded to group allocation."; "We found no difference between groups for age, sex, body weight, or BMI at baseline. However, LDL cholesterol and total fat intake differed at baseline between groups." No issues with the randomization process, however, the baseline differences may suggest a problem with the randomization process
Deviations from the intended interventions	Some concerns	No information about deviations from the intervention.
Missing outcome data	Low	Report data from all randomized participants.
Measurement of the outcome	Low	Quote: "Outcome assessors were blinded to group allocation." Appropriate measurement of outcome was used. Measurement standardized across groups. Although the researchers were not blinded to participant group allocation. However, the assessment of the outcome may not be influenced by the knowledge of the intervention received.
Selection of the reported result	Low	Results were reported in line with the proposed data analysis outlined in the article, and all data reported to be collected was reported in full.
Overall Bias	Some concerns	There are some concerns about the randomization process, and the deviations from the intended interventions, but no issues in relation to any of the other domains assessed.

Title: Additional effect of exercise to intermittent fasting on body composition and cardiometabolic health in adults with overweight/obesity: A systematic review and meta-analysis
First author: Zi-han Dai

Cooke et al., 2022

Risk of bias		
Bias	Authors' judgment	Support for judgment
Randomization process	Some concerns	Quote: "Participants were randomly allocated on a 1:1:1 basis to either 16 weeks of twice weekly fasting (5:2) diet, three sessions a week of SIT, or a combination of the two protocols. Block randomization was used in an attempt to match groups based on baseline BMI, age, predictive daily calorie intake, and physical activity levels."<br>No issues with the randomization process, and the baseline difference, however, it is not known if the allocation sequence was concealed until participants were enrolled and assigned to interventions.
Deviations from the intended interventions	Some concerns	No information about deviations from the intervention.
Missing outcome data	Low	Report data from all randomized participants.
Measurement of the outcome	Some concerns	No information about if the researchers were blinded to participant group allocation. For the body composition outcomes and biomarker outcomes, the measurement using standardized measures is relatively robust. The assessment of these outcomes may not be influenced by the knowledge of the intervention received. However, regarding the aerobic capacity testing, there is no information about if the assessment of the outcome may be influenced by researchers or participants.
Selection of the reported result	Low	Results were reported in line with the proposed data analysis outlined in the article, and all data reported to be collected was reported in full.
Overall Bias	Some concerns	There are some concerns about the randomization process, the deviations from the intended interventions, and the measurement of the outcomes, but no issues concerning any of the other domains assessed.

Title: Additional effect of exercise to intermittent fasting on body composition and cardiometabolic health in adults with overweight/obesity: A systematic review and meta-analysis
First author: Zi-han Dai

Ezpeleta et al., 2023

Risk of bias		
Bias	Authors' judgment	Support for judgment
Randomization process	Low	Quote: "Participants were randomized in a 1:1:1:1 ratio to one of four intervention groups: ADF combined with exercise, ADF alone, exercise alone, or no-intervention control group. Randomization was performed by a stratified random sampling procedure by sex, Age, BMI and IHTG content." "At baseline, there were no significant differences between groups for the primary outcome measure (IHTG content) or any secondary outcome measure." No issues with the randomization process.
Deviations from the intended interventions	Some concerns	Quote: "Due to the nature of the interventions, the study could not be blinded." Participants are aware of the intervention assignment. No information about deviations from the intervention.
Missing outcome data	Low	Quote: "A linear mixed model was used to assess time, group, and time*group effects for each outcome. Linear mixed models for longitudinal data analysis account for missing outcome data using maximum likelihood principles. Thus, these models provide unbiased estimates of time and treatment effects under a missing at-random assumption."
Measurement of the outcome	Low	Quote:" However, study staff involved in outcome ascertainment were blinded as to the subjects." For the body composition outcomes and biomarker outcomes, the measurement using standardized measures is relatively robust. Although theoretically, the recorded measures could be influenced by knowledge of the intervention, this is highly unlikely.
Selection of the reported result	Low	Results were reported in line with the proposed data analysis outlined in the article, and all data reported to be collected was reported in full.
Overall Bias	Some concerns	Some concerns about the deviations from the intended interventions, but no issues about any of the other domains assessed.

Title: Additional effect of exercise to intermittent fasting on body composition and cardiometabolic health in adults with overweight/obesity: A systematic review and meta-analysis
First author: Zi-han Dai

Haganes et al., 2022

Risk of bias		
Bias	Authors' judgment	Support for judgment
Randomization process	Low	Quote: "Participants were randomized 1:1:1:1 to TRE, HIIT, a combination (TREHIIT), or CON, using a random number generator (The Unit for Applied Clinical Research, NTNU, Trondheim). The principal investigator (Dr. Trine Moholdt) performed the randomization of each participant after completed laboratory pre-assessments."; "One week of baseline measurements commenced directly after completed laboratory pre-assessments and randomization, during which participants were instructed to continue with their habitual dietary and physical activity pattern<br>before initiating the assigned protocol on day eight."<br>No issues with the randomization process.
Deviations from the intended interventions	Some concerns	Quote: "Neither the participants nor the study investigators were blinded for group allocation." Participants are aware of the intervention assignment. No information about deviations from the intervention.
Missing outcome data	Low	Report data from all randomized participants.
Measurement of the outcome	Low	For the body composition outcomes and biomarker outcomes, the measurement using standardized measures is relatively robust. Although theoretically, the recorded measures could be influenced by knowledge of the intervention, this is highly unlikely.
Selection of the reported result	Low	Results were reported in line with the proposed data analysis outlined in the article, and all data reported to be collected was reported in full.
Overall Bias	Some concerns	Some concerns about the deviations from the intended interventions, but no issues in relation to any of the other domains assessed.

Title: Additional effect of exercise to intermittent fasting on body composition and cardiometabolic health in adults with overweight/obesity: A systematic review and meta-analysis
First author: Zi-han Dai

Keawtep et al., 2024

Risk of bias		
Bias	Authors' judgment	Support for judgment
Randomization process	Some concerns	Quote: "The study was a 3-month, assessor-blinded, four-arm randomized controlled trial. Permuted block randomization (random block sizes) was used to allocate all participants into four groups with a 1:1:1:1 ratio using a computer-generated random number sequence."<br>No issues with the randomization process. No baseline differences that suggested any issues with randomization. However, regarding the allocation sequence concealed until participants were enrolled and assigned to intervention is unclear.
Deviations from the intended interventions	Some concerns	No information about deviations from the intervention.
Missing outcome data	Low	Report data from all randomized participants.
Measurement of the outcome	Low	The study was assessor-blinded, appropriate measurement of outcome was used. Measurement standardized across groups. Although the researchers were not blinded to participant group allocation. However, the assessment of the outcome may not be influenced by the knowledge of the intervention received.
Selection of the reported result	Low	The data reported were in accordance with the prespecified protocol, published within the authors' organization.
Overall Bias	Some concerns	There are some concerns about the randomization process, and the deviations from the intended interventions, but no issues in relation to any of the other domains assessed.

Title: Additional effect of exercise to intermittent fasting on body composition and cardiometabolic health in adults with overweight/obesity: A systematic review and meta-analysis
First author: Zi-han Dai

Maaloul et al., 2022

Risk of bias		
Bias	Authors' judgment	Support for judgment
Randomization process	High	Quote: "The study was conducted during 30 RDIF days. Participants were randomized into 2 groups" However, no information regarding the baseline difference, and regarding the allocation sequence concealed until participants were enrolled and assigned to intervention is unclear.
Deviations from the intended interventions	Some concerns	Participants and investigators are aware of the intervention assignment. No information about deviations from the intervention.
Missing outcome data	Low	Report data from all randomized participants.
Measurement of the outcome	Low	For the body composition outcomes and biomarker outcomes, the measurement using standardized measures is relatively robust. Although theoretically, the recorded measures could be influenced by knowledge of the intervention, this is highly unlikely.
Selection of the reported result	Some concerns	Although results were reported in line with the article method part, the proposed data analysis cannot be found.
Overall Bias	High	Some concerns about the deviations from the intended interventions, and selection of the reported result; as high bias regarding the randomization process, while no issues about the other domains assessed.

Title: Additional effect of exercise to intermittent fasting on body composition and cardiometabolic health in adults with overweight/obesity: A systematic review and meta-analysis
First author: Zi-han Dai

Oh et al., 2018

Risk of bias		
Bias	Authors' judgment	Support for judgment
Randomization process	Low	Quote: "Permuted-block randomization was used to allocate all participants into 4 groups in a 1:1:1:1 ratio using a computer-generated random number sequence."; "There is no significant difference between groups in age, height, weight, BMI, WC, level of blood pressure, and insulin resistance" No issues with the randomization process. No baseline differences that suggested any issues with randomization.
Deviations from the intended interventions	Some concerns	Quote: "First, although a double-blind design was initially implemented to elicit unpolluted results and to prevent any bias, the participants became aware of their respective groups during the diet and exercise education orientation." Participants and the carers may be aware of the interventions. No information about deviations from the intervention.
Missing outcome data	Low	Report data from all randomized participants.
Measurement of the outcome	Low	Quote: "Additionally, all of the trained exercise specialists who took the measurements were blinded to group allocation." For the body composition outcomes and biomarker outcomes, the measurement using standardized measures is relatively robust. Although theoretically, the recorded measures could be influenced by knowledge of the intervention, this is highly unlikely.
Selection of the reported result	Low	Results were reported in line with the proposed data analysis outlined in the article, and all data reported to be collected was reported in full.
Overall Bias	Some concerns	There are some concerns about the deviations from the intended interventions, but no issues with any of the other domains assessed.

Title: Additional effect of exercise to intermittent fasting on body composition and cardiometabolic health in adults with overweight/obesity: A systematic review and meta-analysis
First author: Zi-han Dai

Table S4. Overall certainty of evidence

Certainty assessment							№ of patients		Effect		Certainty	Importance
№ of studies	Study design	Risk of bias	Inconsistency	Indirectness	Imprecision	Other considerations	IF+EX	IF	Relative (95% CI)	Absolute (95% CI)		
**Body mass (assessed with: kg)**												
10	randomised trials	serious[a]	not serious	not serious	serious[b]	publication bias strongly suspected[c]	163	156	-	MD **0.15 kg lower** (0.84 lower to 0.54 higher)	⊕◯◯◯ Very low[a,b,c]	
**BMI (assessed with: kg/m^2)**												
7	randomised trials	serious[a]	not serious	not serious	serious[b]	none	115	109	-	MD **0.26 kg/m^2 lower** (0.67 lower to 0.15higher)	⊕⊕◯◯ Low[a,d]	
**Fat mass (assessed with: kg)**												
10	randomised trials	serious[a]	not serious	not serious	not serious	none	163	156	-	MD **0.93 kg lower** (1.69 lower to 0.18 lower)	⊕⊕⊕◯ Moderate[a]	
**Fat-free mass (assessed with: kg)**												
9	randomised trials	serious[a]	serious[e]	not serious	serious[b]	none	154	148	-	MD **0.75 kg higher** (0.41 lower to 1.9 higher)	⊕◯◯◯ Very low[a,b,e]	
**Waist circumference (assessed with: cm)**												
7	randomised trials	serious[a]	not serious	not serious	serious[d]	none	104	96	-	MD **2.51 cm lower** (3.7 lower to 1.32 lower)	⊕⊕◯◯ Low[a,d]	

Title: Additional effect of exercise to intermittent fasting on body composition and cardiometabolic health in adults with overweight/obesity: A systematic review and meta-analysis
First author: Zi-han Dai

Certainty assessment							№ of patients		Effect		Certainty	Importance
№ of studies	Study design	Risk of bias	Inconsistency	Indirectness	Imprecision	Other considerations	IF+EX	IF	Relative (95% CI)	Absolute (95% CI)		
**Visceral fat mass**												
4	randomised trials	serious[a]	not serious	not serious	serious[d]	none	67	64	-	SMD **0.2 SD lower** (0.55 lower to 0.15 higher)	⨁⨁◯◯ Low[a,d]	
**Fasting glucose (assessed with: mg/dl)**												
9	randomised trials	serious[a]	serious[e]	not serious	serious[b]	none	148	145	-	MD **1.92 mg/dl lower** (4.47 lower to 0.62 higher)	⨁◯◯◯ Very low[a,b,e]	
**Insulin (assessed with: uIU/ml)**												
7	randomised trials	serious[a]	not serious	not serious	serious[d]	none	126	124	-	MD **3.1 uIU/ml lower** (4.25 lower to 1.95 lower)	⨁⨁◯◯ Low[a,d]	
**HOMA-IR**												
8	randomised trials	serious[a]	not serious	not serious	serious[d]	none	137	132	-	SMD **0.57 SD lower** (0.83 lower to 0.31 lower)	⨁⨁◯◯ Low[a,d]	
**Total cholesterol (assessed with: mg/dl)**												
9	randomised trials	serious[a]	serious[e]	not serious	serious[b]	none	147	143	-	MD **7.39 mg/dl lower** (16.53 lower to 1.74 higher)	⨁◯◯◯ Very low[a,b,e]	

Title: Additional effect of exercise to intermittent fasting on body composition and cardiometabolic health in adults with overweight/obesity: A systematic review and meta-analysis
First author: Zi-han Dai

Certainty assessment							№ of patients		Effect		Certainty	Importance
№ of studies	Study design	Risk of bias	Inconsistency	Indirectness	Imprecision	Other considerations	IF+EX	IF	Relative (95% CI)	Absolute (95% CI)		
**High-density lipoprotein cholesterol (assessed with: mg/dl)**												
8	randomised trials	serious[a]	serious[e]	not serious	serious[d]	none	124	120	-	MD **3.32 mg/dl higher** (1.56 lower to 8.21 higher)	⨁◯◯◯ Very low[a,d,e]	
**Low-density lipoprotein cholesterol (assessed with: mg/dl)**												
7	randomised trials	serious[a]	serious[e]	not serious	serious[d]	none	112	107	-	MD **10.67 mg/dl lower** (20 lower to 1.35 lower)	⨁◯◯◯ Very low[a,d,e]	
**Triglycerides (assessed with: mg/dl)**												
9	randomised trials	serious[a]	serious[e]	not serious	serious[b]	none	147	143	-	MD **12.86 mg/dl lower** (34.11 lower to 8.4 higher)	⨁◯◯◯ Very low[a,b,e]	
**SBP (assessed with: mmHg)**												
6	randomised trials	serious[a]	serious[e]	not serious	serious[d]	none	105	104	-	MD **0.16 mmHg higher** (2.66 lower to 2.99 higher)	⨁◯◯◯ Very low[a,d,e]	
**DBP (assessed with: mmHg)**												
6	randomised trials	serious[a]	serious[e]	not serious	serious[d]	none	105	104	-	MD **0.05 mmHg lower** (1.98 lower to 1.88 higher)	⨁◯◯◯ Very low[a,d,e]	

Title: Additional effect of exercise to intermittent fasting on body composition and cardiometabolic health in adults with overweight/obesity: A systematic review and meta-analysis
First author: Zi-han Dai

Certainty assessment							№ of patients		Effect		Certainty	Importance
№ of studies	Study design	Risk of bias	Inconsistency	Indirectness	Imprecision	Other considerations	IF+EX	IF	Relative (95% CI)	Absolute (95% CI)		
**Resting heart rate (assessed with: bpm)**												
3	randomised trials	serious[a]	not serious	not serious	serious[d]	none	62	63	-	MD **2.68 bpm lower** (4.71 lower to 0.64 lower)	⨁⨁◯◯ Low[a,d]	
**VO_{2max} (assessed with: ml/kg/min)**												
5	randomised trials	serious[a]	serious[e]	not serious	serious[d]	none	70	66	-	MD **1.80 mg/kg/min higher** (0.12 higher to 3.48 higher)	⨁◯◯◯ Very low[a,d,e]	
**Leptin (assessed with: ng/ml)**												
2	randomised trials	serious[a]	not serious	not serious	serious[d]	none	41	41	-	MD **13.76 ng/ml lower** (25.76 lower to 1.76 lower)	⨁⨁◯◯ Low[a,d]	
**Adiponectin**												
3	randomized trials	serious[a]	not serious	not serious	serious[d]	none	63	63	-	SMD **0.35 SD higher** (0 to 0.7 higher)	⨁⨁◯◯ Low[a,d]	

Note: BMI, body mass index; CI, confidence interval; DBP, diastolic blood pressure; HOMA-IR, homeostatic model assessment for insulin resistance; MD, mean difference; SBP, systolic blood pressure; SD, standard deviation; SMD, standardized mean difference; VO_{2max}, maximal oxygen uptake

Explanations

a. Most of the included studies exhibited some concerns in the risk of bias assessment, which may have an impact on the certainty of the findings.

b. Imprecise due to confidence intervals included potential for important harm or benefit.

c. Funnel plot display asymmetry, which may raise concerns regarding the potential presence of publication bias.

d. Small sample size

e. I^2 values showed high heterogeneity

Title: Additional effect of exercise to intermittent fasting on body composition and cardiometabolic health in adults with overweight/obesity: A systematic review and meta-analysis
First author: Zi-han Dai

Table S5. Quality of the included studies using the Tool for the assEssment of Study qualiTy and reporting in Exercise (TESTEX) scale.

	Study quality					Score	Study reporting										Score	Total score
**Study**	**1**	**2**	**3**	**4**	**5**	**(0-5)**	**6a**	**6b**	**6c**	**7**	**8a**	**8b**	**9**	**10**	**11**	**12**	**(0-10)**	**(0-15)**
Ameur et al., 2024	1	1	0	1	0	3	1	0	0	0	1	1	1	0	1	0	5	8
Batitucci et al., 2022	1	1	0	0	1	3	0	0	0	0	1	1	1	0	1	1	5	8
Batitucci et al., 2024	1	1	0	0	0	2	0	0	0	0	1	1	1	0	1	1	5	7
Bhutani et al., 2013a	1	1	0	0	0	2	0	0	0	1	1	1	1	0	1	1	6	8
Bhutani et al., 2013b	1	1	0	0	0	2	0	0	0	1	1	1	1	0	1	1	6	8
Cho et al., 2019	0	1	1	1	1	4	0	0	0	0	1	1	1	1	1	0	5	9
Cooke et al., 2022	0	0	0	0	0	0	0	0	0	1	1	1	1	1	1	0	6	6
Ezpeleta et al., 2023	1	1	0	1	1	4	1	0	1	1	1	1	1	1	1	1	9	13
Haganes et al., 2022	1	1	1	1	0	4	0	0	0	1	1	1	1	0	1	1	6	10
Keawtep et al., 2024	1	1	0	1	1	4	1	1	1	1	1	1	1	0	1	0	8	12
Maaloul et al., 2023	1	0	0	1	0	2	1	0	0	1	1	1	1	0	1	1	7	9
Oh et al., 2018	1	1	0	0	1	3	0	0	0	1	1	1	1	0	1	1	6	9

Study quality: 1 = Eligibility criteria specified; 2 = Randomization specified; 3 = Allocation concealment; 4 = Groups similar at baseline; 5 = Blinding of assessor (for at least one key outcome). Study reporting: 6 = Outcome measures assessed in 85% of participants (6a = 1 point if completion rate is >85%; 6b = 1 point if adverse events are reported; 6c = 1 point if exercise attendance is reported); 7 = Intention-to-treat analysis; 8 = Between-group statistical comparisons reported (8a = 1 point if between-group statistical comparisons are reported for the primary outcome measure of interest; 8b = 1 point if between-group statistical comparisons are reported for at least one secondary outcome measure); 9 = Point measures and measures of variability for all reported outcome measures; 10 = Activity monitoring in control groups; 11 = Relative exercise intensity remained constant; 12 = All exercise characteristics are reported adequately (i.e. intensity, frequency, mode, duration of session and duration of the intervention). The studies were classified according to their total TESTEX score as 'high quality' (≥ 12 points, green colour), 'good quality' (7 to 11 points, yellow colour), or 'low quality' (≤ 6 points, red colour).

Title: Additional effect of exercise to intermittent fasting on body composition and cardiometabolic health in adults with overweight/obesity: A systematic review and meta-analysis
First author: Zi-han Dai

Table S6. Funnel plot for publication bias detection on body mass and fat mass

Body mass: P-value = 0.0023

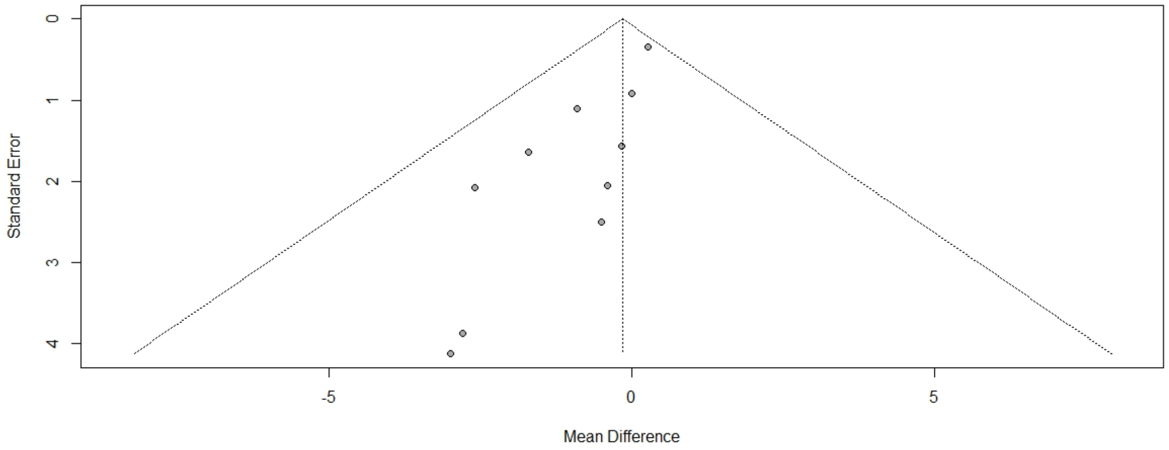

Fat mass: P-value = 0.0617

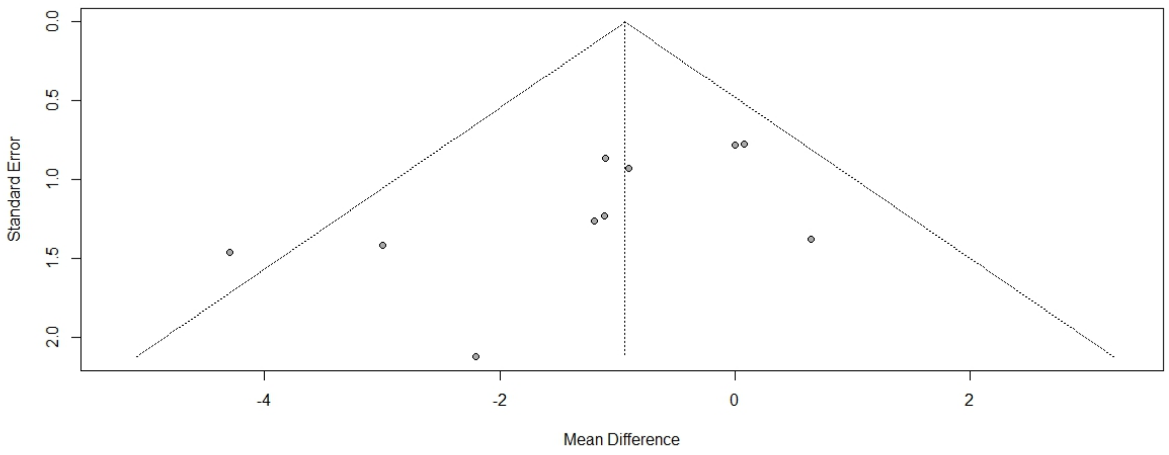

Title: Additional effect of exercise to intermittent fasting on body composition and cardiometabolic health in adults with overweight/obesity: A systematic review and meta-analysis
First author: Zi-han Dai

Figure S1. Meta-analysis of IF+EX vs. IF alone on body mass. Values are reported as mean difference (MD).

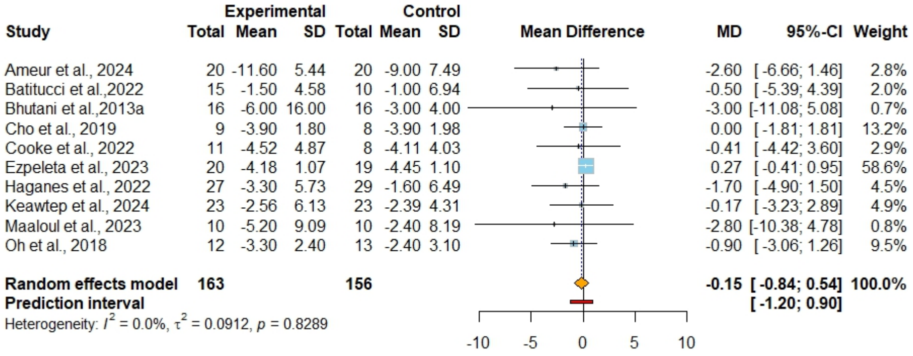

Figure S2. Meta-analysis of IF+EX vs. IF alone on body mass index (BMI). Values are reported as mean difference (MD).

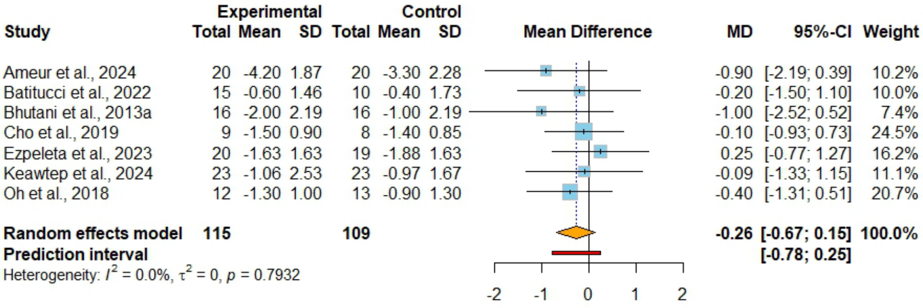

Figure S3. Meta-analysis of IF+EX vs. IF alone on fat-free mass. (MD)

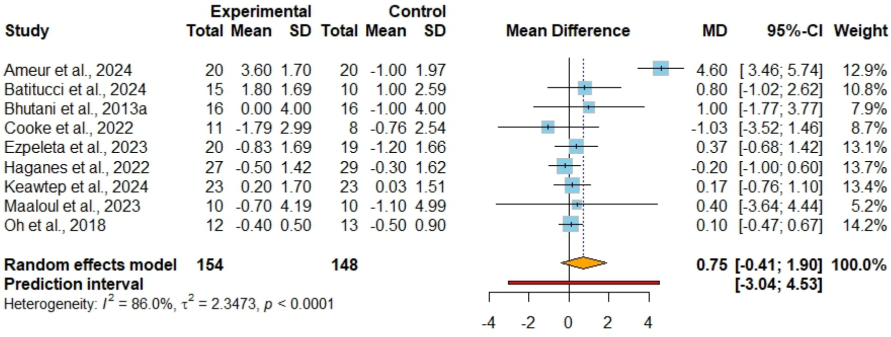

Title: Additional effect of exercise to intermittent fasting on body composition and cardiometabolic health in adults with overweight/obesity: A systematic review and meta-analysis
First author: Zi-han Dai

Figure S4. Meta-analysis of IF+EX vs. IF alone on visceral fat, presented as standardized mean differences (SMD).

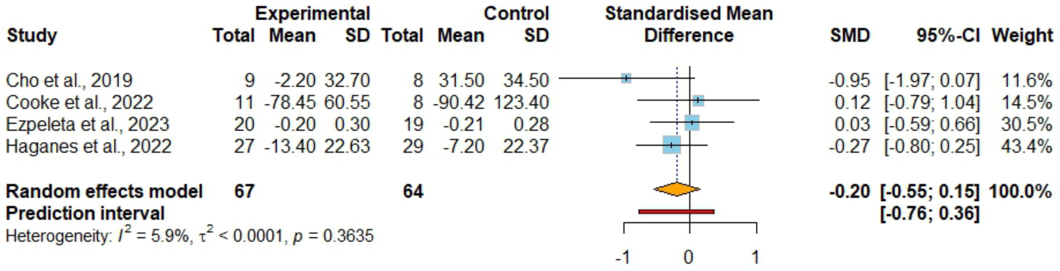

Figure S5. Meta-analysis of IF+EX vs. IF alone on fasting glucose. (MD)

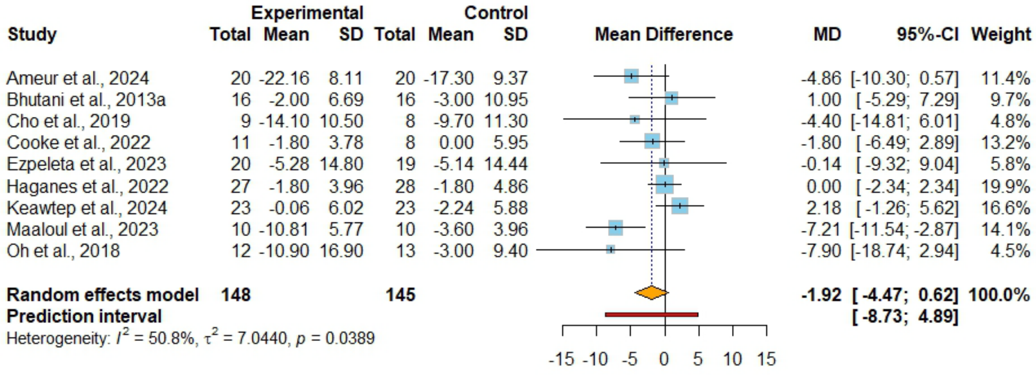

Figure S6. Meta-analysis of IF+EX vs. IF alone on total cholesterol. (MD)

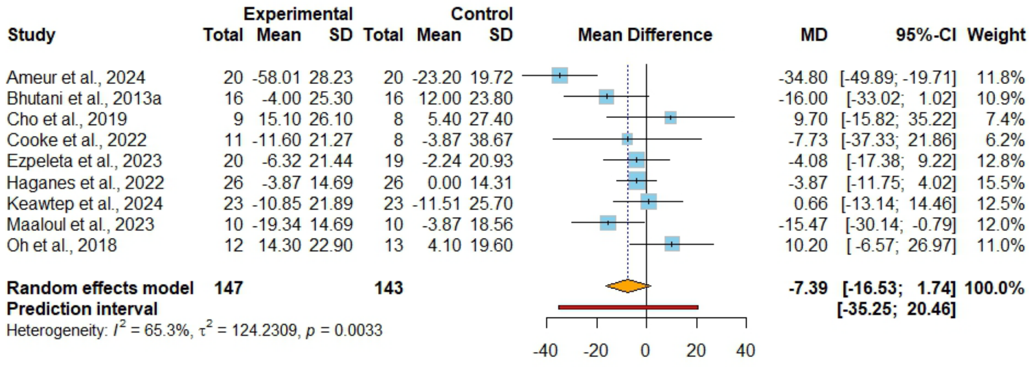

Title: Additional effect of exercise to intermittent fasting on body composition and cardiometabolic health in adults with overweight/obesity: A systematic review and meta-analysis
First author: Zi-han Dai

Figure S7. Meta-analysis of IF+EX vs. IF alone on high-density lipoprotein cholesterol. (MD)

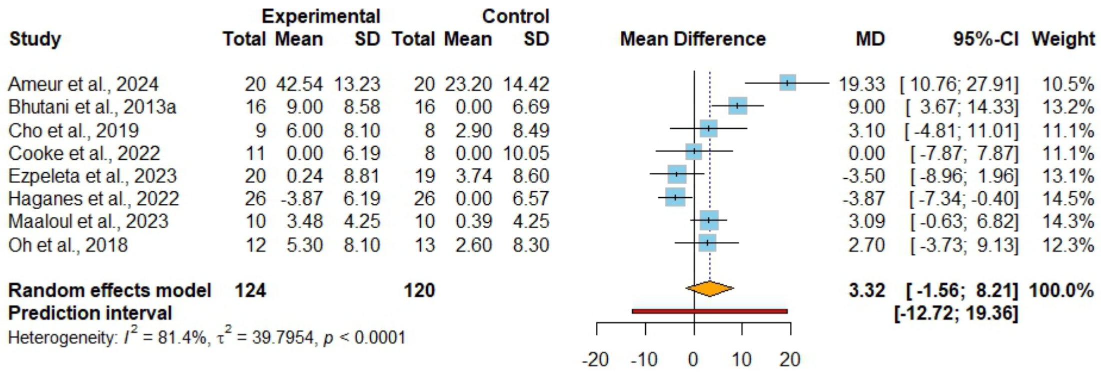

Figure S8. Meta-analysis of IF+EX vs. IF alone on triglycerides. (MD)

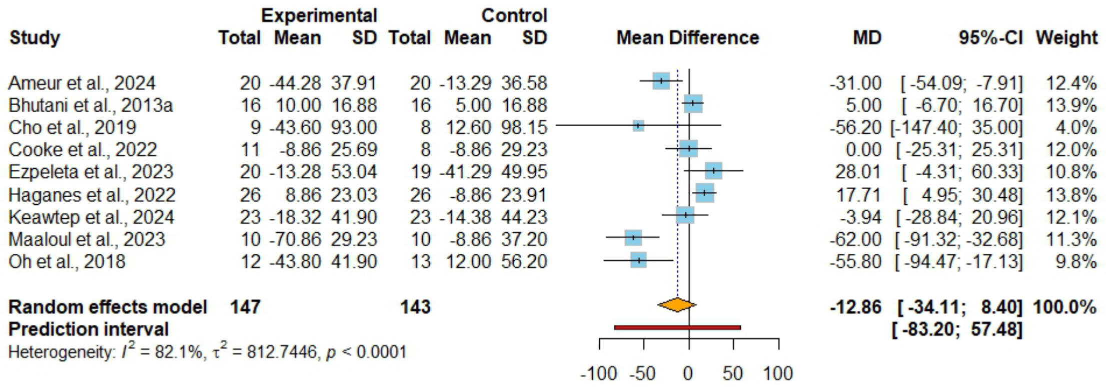

Figure S9. Meta-analysis of IF+EX vs. IF alone on systolic blood pressure. (MD)

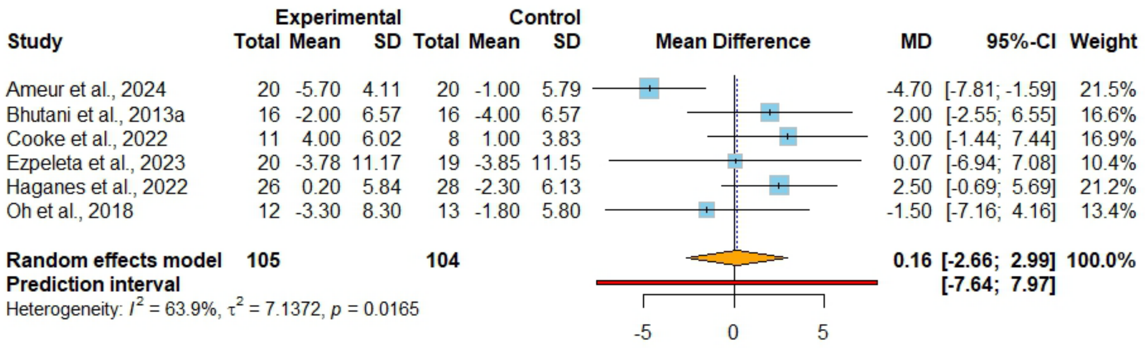

Title: Additional effect of exercise to intermittent fasting on body composition and cardiometabolic health in adults with overweight/obesity: A systematic review and meta-analysis
First author: Zi-han Dai

Figure S10. Meta-analysis of IF+EX vs. IF alone on diastolic blood pressure. (MD)

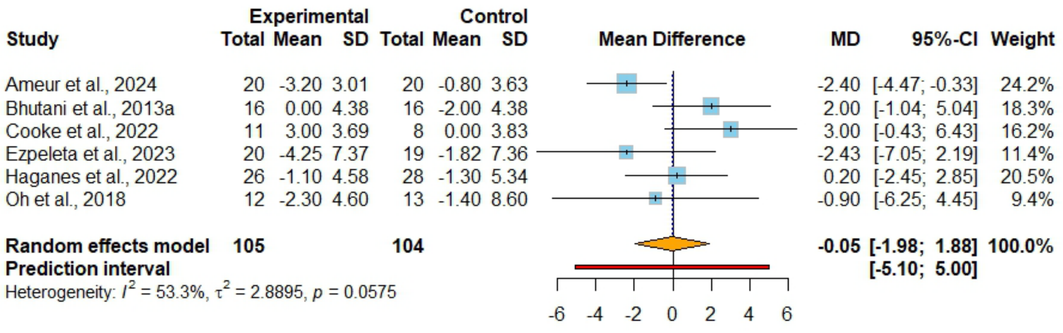

Figure S11. Meta-analysis of IF+EX vs. IF alone on leptin. (MD)

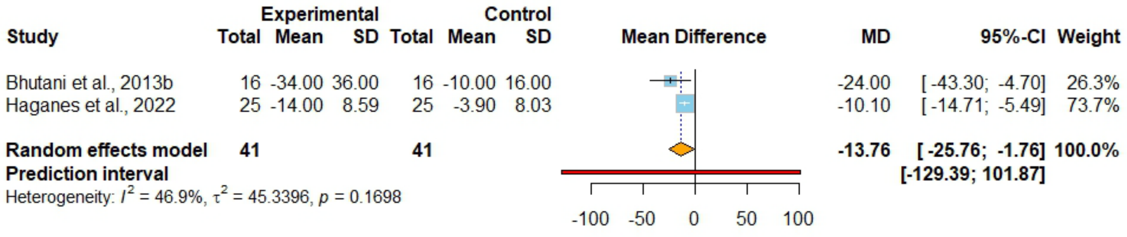

Figure S12. Meta-analysis of IF+EX vs. IF alone on adiponectin. (SMD)

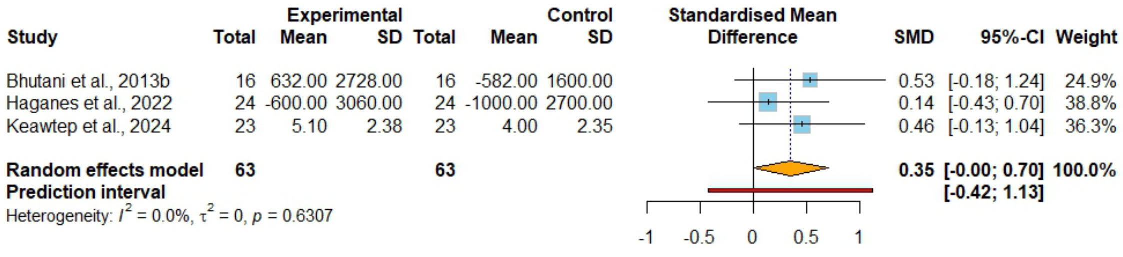

Supplement: Supplementary file 1 — Supplementary file1 (PDF 2768 KB) [file 13679_2025_645_MOESM1_ESM.pdf]
